# Efficient Cu-catalyzed base-free C–S coupling under conventional and microwave heating. A simple access to S-heterocycles and sulfides

**DOI:** 10.3762/bjoc.9.50

**Published:** 2013-03-04

**Authors:** Silvia M Soria-Castro, Alicia B Peñéñory

**Affiliations:** 1 INFIQC, Departamento de Química Orgánica, Facultad de Ciencias Químicas, Universidad Nacional de Córdoba, Ciudad Universitaria, X5000HUA. Córdoba, Argentina, Fax: (+54) 351-4333030

**Keywords:** catalysis, copper, cross-coupling, potassium thioacetate, sulfur heterocycles

## Abstract

*S*-aryl thioacetates can be prepared by reaction of inexpensive potassium thioacetate with both electron-rich and electron-poor aryl iodides under a base-free copper/ligand catalytic system. CuI as copper source affords *S*-aryl thioacetates in good to excellent yields, by using 1,10-phenanthroline as a ligand in toluene at 100 °C after 24 h. Under microwave irradiation the time was drastically reduced to 2 h. Both procedures are simple and involve a low-cost catalytic system. This methodology was also applied to the “one-pot” synthesis of target heterocycles, such as 3*H*-benzo[*c*][1,2]dithiol-3-one and 2-methylbenzothiazole, alkyl aryl sulfides, diaryl disulfides and asymmetric diaryl sulfides in good yields.

## Introduction

**Figure 1:**
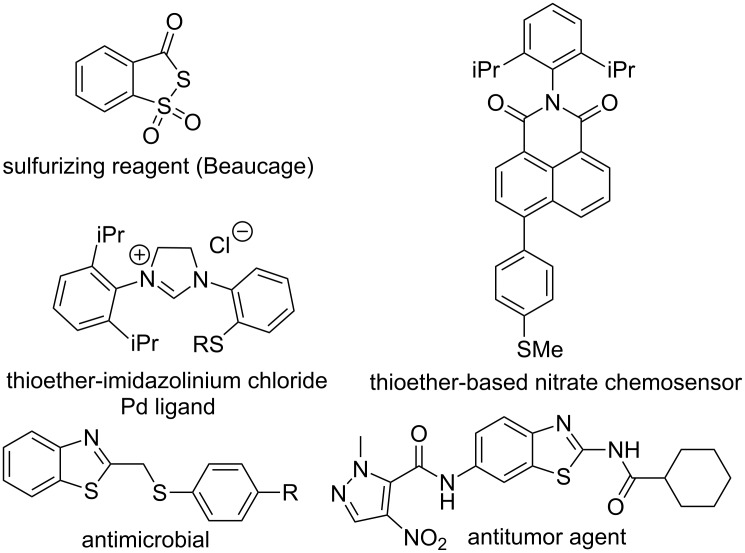
Examples of some pharmaceutically and chemically important derivatives.

Aromatic sulfur compounds including sulfides and heterocycles are relevant in chemical, pharmaceutical and materials industries [1–3]. As a consequence, the development of new methodologies for C_Ar_–S bond formation still represents a challenge in organic chemistry (Figure 1).

Nucleophilic substitution of aryl halides by a variety of sulfur anions, such as PhS^−^, 1-naphthylS^−^, S^2−^, thiourea anion (^−^SCNH(NH_2_)), thioacetate anion (MeCOS^−^) and thiobenzoate anion (PhCOS^−^), can be effectively achieved by photoinduced electron transfer reactions to form new C–S bonds [4–5]. We have previously described the reactivity of sulfur anions, such as S^2−^ [6], ^−^SCNH(NH_2_) [7–8], and MeCOS^−^ [9], in photoinduced aromatic radical nucleophilic substitution (S_RN_1 mechanism), as a “one-pot” method for the synthesis of several sulfur aromatic compounds in moderate to good yields. A common feature of these reactions concerns the formation of arylthiolate anions as intermediates, offering a viable alternative for the introduction of sulfur functionalities in aryl compounds.

The formation of C–S bonds can also be accomplished in good to excellent yields by transition-metal catalysis [10–11], mainly using palladium [12], copper [13–16], nickel [17–18] and iron [19–20] salts. Aryl coupling reactions employing different palladium species as catalysts represent the most extensively studied approaches to form new C_Ar_–S bonds [21–23]. Nevertheless, copper or iron-mediated coupling reactions have become a convenient alternative to the expensive Pd/ligand systems due to the lower cost of the former, its stability, and the ready availability of the ligands. Thus, different copper-salt-based catalytic systems have been found to be effective for the aryl coupling reactions of ArSH [24–29], RSO_2_Na [30], KSCN [31–32], NH_2_CSNH_2_ [33–34], MeCSNH_2_ [35], KSCSOEt [36–37], Na_2_S·9H_2_O [38–39] and sulfur [40–41] as well as the synthesis of diverse heterocycles containing sulfur atoms by intra- [42–44] and intermolecular [45–47] reactions.

Aryl thioesters are versatile intermediates for the synthesis of a variety of sulfur compounds including aryl thiols, aryl alkyl sulfides, aryl sulfenyl chlorides, aryl sulfonyl chlorides [48] and sulfonamides [49]. Thioacetate and thiobenzoate derivatives have been synthesized by the reactions of thioacetate and thiobenzoate anions with arenediazonium tetrafluoroborates [50]. However, this methodology implies moderate overall yields and the handling of usually unstable diazonium salts. More recently, *S-*aryl thioacetates have been obtained by the catalyst system [Pd_2_(dba)_3_-Xantphos] in 1,4-dioxane under microwave heating at 160 °C in good yields [51]. The synthesis of arylthiobenzoates by a CuI/1,10-phenanthroline, starting from thiobenzoic acid and iPr_2_NEt (DIPEA) in toluene after 24 h at 110 °C, has also been reported [52].

As part of our ongoing study on sulfur chemistry, we are interested in the one-pot synthesis of target heterocycles such as the Beaucage reagent and benzothiazole as well as sulfides by using Cu-catalysis to mediate the C–S bond formation. Herein, we report the reactions of KSCOMe (**1**) with aryl iodides under a Cu/ligand/base-free system to afford *S*-aryl thioacetates **2** as thiol surrogates in good to excellent yields, under conventional heating and microwave irradiation as an efficient access to a variety of sulfur compounds.

## Results and Discussion

**Scheme 1:**
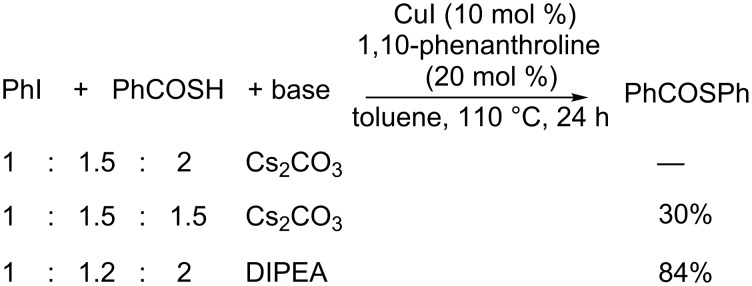
Synthesis of *S*-phenyl benzothioate.

Initially, we tried PhCOSH with PhI using the catalytic systems of CuI/1,10-phenanthroline in toluene according to the method described by Sawada [52]. This methodology proved particularly sensitive to the nature and concentration of the base used in the deprotonation of the phenyl thiobenzoic acid due to an undesired hydrolysis of the thioester primary product. Thus, the reaction with a PhI/PhCOSH/Cs_2_CO_3_ ratio of 1:1.5:2 afforded Ph_2_S without traces of the thioester, and with a ratio of 1:1.5:1.5 the PhSCOPh was isolated in only 30% yield. Finally, the formation of PhSCOPh in our hands increased to 84% (quantified by GC) by using DIPEA as base with a PhI/PhCOSH/DIPEA ratio of 1:1.2:2 (Sawada’s conditions) [52] (Scheme 1).

**Scheme 2:**
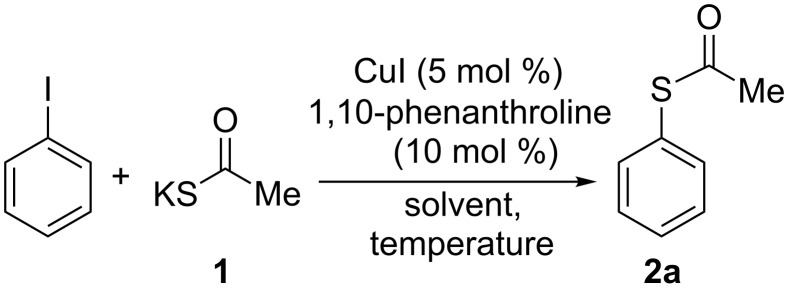
Synthesis of *S*-phenyl thioacetate.

Considering that the commercially available salt KSCOMe (**1**) is a solid, is easy to handle, is less odorous, and avoids the use of a relative expensive base with a lesser impact on the environment, as compared with the liquid PhCOSH, we continued our study on the synthesis of sulfides by using salt **1**. The model reaction of PhI with KSCOMe was selected to determine the optimal conditions (solvent and temperature) starting with the effective CuI and 1,10-phenanthroline catalytic system previously employed (Scheme 2).

The use of the polar solvents DMF, MeCN and DMSO gave no conversion at all or yielded only a trace amount of *S*-phenyl thioacetate (**2a**) together with Ph_2_S and Ph_2_S_2_ from the in situ generation of benzenethiolate anion (Table 1, entries 1–3). In addition, no reaction in THF occurred after 48 h of heating under reflux, whereas **2a** was formed in low yield when the reaction was performed in 1,4-dioxane, (Table 1, entries 4 and 5). Conversely, better results were obtained in nonpolar toluene at 100 °C affording 34% isolated yield of **2a** after 24 h. This coupling reaction did not occur under air or in the absence of the ligand and was improved to 96% yield by using 10 and 20 mol % of CuI salt and ligand, respectively, (Table 1, entries 6–9). Similarly, a series of ligands usually employed in copper-catalyzed cross-coupling reactions was also screened, such as L-proline, benzotriazole, tetramethylethylenediamine (TMEDA), dimethylethylenediamine (DMEDA), and acetylacetone, under the optimized reaction conditions with 1,10-phenanthroline (Table 1, entry 9). These ligands, with different coordinating and structural properties, gave no positive results, and PhI was quantitatively recovered.

**Table 1:** Screening of solvents for the CuI-catalyzed reaction of potassium thioacetate (**1**) with iodobenzene.^a^

entry	solvent	temp. [°C]	time [h]	**2a**, yield (%)^b^

1	DMF	80	24	0
2	MeCN	80	39	<5^c^
3	DMSO	100	24	<5^c^
4	THF	67	48	0
5	Dioxane	100	24	6
6	Toluene	100	24	34^d^
7^e^	Toluene	100	24	0
8^f^	Toluene	100	24	0
**9^g^**	**Toluene**	**100**	**24**	**96**

^a^Reaction conditions: CuI (5 mol %, 0.025 mmol), 1,10-phenanthroline (10 mol %, 0.05 mmol), **1** (0.75 mmol), PhI (0.5 mmol), solvent (4 mL), conventional heating. ^b^Quantified by GC by the internal standard method. ^c^Together with Ph_2_S and Ph_2_S_2_ as main products. ^d^Isolated yield. ^e^Under an air atmosphere. ^f^In the absence of ligand. ^g^CuI (10 mol %), 1,10-phenanthroline (20 mol %).

The effect of the Cu(I) and Cu(II) salts on the coupling reaction was also evaluated, and the results are summarized in Table 2. CuI and CuCl exhibited similar catalytic activity as did Cu(II) chloride dihydrate (Table 2, entries 1–3). There is general agreement that Cu(I) is the catalytic species in copper-catalyzed arylation of C, N or O-nucleophiles, which can be generated from Cu(II) or Cu(0) precursors by an in situ reduction or oxidation, respectively [53–55]. On the other hand, OTf^−^ or OAc^−^ counter ions decrease catalytic activity in Cu(II) salts, in comparison to chloride ions (Table 2, entries 4 and 5). Catalysis by CuO also affords moderate yield (Table 2, entry 6). Finally, in the absence of a copper source, no reaction occurred, supporting the participation of the copper species in the coupling reaction between thioacetate ion and PhI (Table 2, entry 7).

**Table 2:** Screening of copper sources for the copper-catalyzed reaction of potassium thioacetate (**1**) with iodobenzene.^a^

entry	[Cu]	**2a**, yield (%)^b^

1	CuI	96
2	CuCl	93
3	CuCl_2_·2H_2_O	87
4	Cu(OAc)_2_	29
5	Cu(OTf)_2_	37
6	CuO	41
7	—	0

^a^Reaction conditions: [Cu] (10 mol %, 0.05 mmol); 1,10-phenanthroline (20 mol %, 0.1 mmol), **1** (0.75 mmol), PhI (0.5 mmol), toluene (4 mL), 100 °C (conventional heating), 24 h. ^b^Quantified by GC by the internal standard method.

A variety of commercially available aryl iodides were subjected to the optimized reaction conditions (Table 2, entry 1) to determine the scope of this catalytic system for the synthesis of *S*-aryl thioacetates **2**, and the results are summarized in Table 3.

**Table 3:** Copper-catalyzed reaction of aryl iodides with potassium thioacetate (**1**).^a^

entry	aryl iodide	product	**2** yield (%)^b^ method	
			A	B

1	PhI	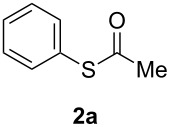	96 (80)	92 (77)
2	*p*-MeC_6_H_4_I	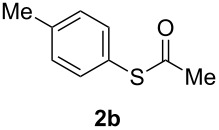	96 (84)	
3	*p*-MeOC_6_H_4_I	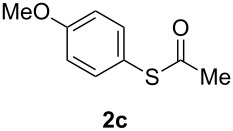	94 (74)	74 (69)
4	*p*-MeCONHC_6_H_4_I	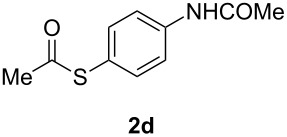	(72)	
5	*p*-MeCOC_6_H_4_I	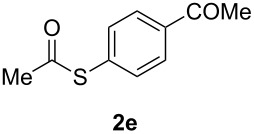	(89)	79 (69)
6	*p*-NO_2_C_6_H_4_I	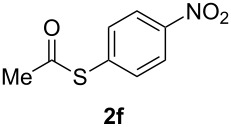	(73)	
7	*p*-CNC_6_H_4_I	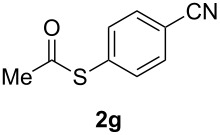	73	64 (58)
8	*o*-MeC_6_H_4_I	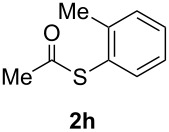	84 (71)	
9	*o*-MeOC_6_H_4_I	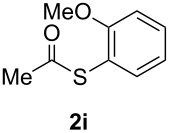	(76)	75 (67)
10	3-C_5_NH_4_I	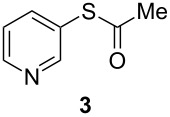	(82)	
11	1-C_10_H_7_I	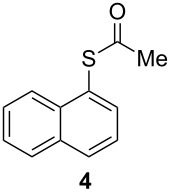	(75)	
12	*p*-C_6_H_4_I_2_	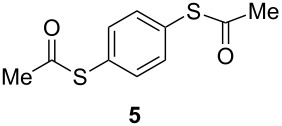	(63)	99 (70)
13	PhBr	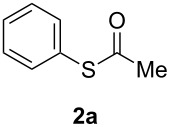	—	—

^a^Reaction conditions: CuI (10 mol %, 0.05 mmol); 1,10-phenanthroline (20 mol %, 0.1 mmol), **1** (0.75 mmol), ArI (0.5 mmol); method A: toluene (4 mL), 100 °C (conventional heating), 24 h; method B: toluene (2 mL), microwave irradiation at 25 W, temperature between 110 °C and 140 °C, 2 h. ^b^Quantified by GC by the internal standard method and isolated yield between parentheses. ^c^ArI_2_:**1** ratio of 1:2.

As described in Table 3, the coupling of thioacetate with aryl iodides bearing electron-withdrawing (EWGs) and electron-donating (EDGs) groups proceeded in good to excellent yields (63–96%). For instance, the *para*-methoxy and *para*-acetyl phenyl iodides afforded thioacetates **2c** and **2e** in 74% and 89% isolated yield, respectively (Table 3, entries 3 and 5). In the absence of the catalytic system CuI/1,10-phenanthroline, the aryl iodides bearing the *para-*acetyl, *para*-nitro and *para*-cyano substituents, did not react with potassium thioacetate (**1**) after 24 h in toluene at 110 °C. These observations rule out the possibility of an aromatic nucleophilic substitution to account for the obtained results.

Furthermore, sterically hindered *ortho-*substituted aryl iodides afforded good yields of the coupling products **2** (Table 3, entries 8 and 9). For example, the *ortho*- and *para*-methoxy aryl iodides rendered comparable isolated yields of thioacetate derivatives **2c** and **2i** (Table 3, entries 3 and 9). The reaction between 4-iodoaniline and **1** under CuI/1,10-phenanthroline catalysis afforded quantitatively the corresponding 4-iodoacetanilide as the only product. The acetylation of the amino group competes effectively with the cross-coupling reaction. A similar acetylation reaction was also observed with 2-iodophenol. In consequence, protected amino and hydroxy groups were required for the copper-mediated C–S bond formation to proceed properly (Table 3, entries 3, 4 and 9).

This copper-catalyzed coupling reaction was also applied to heteroaromatic and other aromatics such as 3-iodopyridine and 1-naphthyl iodide, affording thioacetate derivatives **3** and **4** in good isolated yields. Interestingly, the reaction of 1,4-diiodobenzene with thioacetate rendered the corresponding disubstituted product **5**, a straightforward precursor of 1,4-benzenedithiol, in high isolated yield. As a result, the simple methodology here presented could be an attractive alternative for the synthesis of polysulfides and other sulfur relevant materials (Table 3, entries 10–12).

In a comparative fashion we have also performed these reactions under microwave irradiation in order to improve conversion times and yields (Table 3, Method B). These reactions occur in only 2 h with yields comparable to those under conventional heating, for EWGs and EDGs (Table 3, entries 1, 3, 5, 7, 9 and 12). Bromobenzene was unreactive towards **1** under both conventional and microwave heating (Table 3, entry 13), this Cu-catalyzed C–S coupling reaction being highly chemoselective for aryl iodides.

In comparison with the already reported procedure for the preparation of *S*-aryl thioacetates by Pd catalysis [51,56], the methodology herein described has the advantages of using a lower-cost copper salt and a stable and accessible ligand. Furthermore, the use of a solid air-stable KSCOMe salt instead of a highly inflammable liquid and corrosive PhCOSH and DIPEA [52] offered environmental advantages, due to the absence of any additional base and the ease of handling*.* Moreover the use of microwave irradiation with concomitant decrease of the reaction times to only 2 h is noteworthy.

**Scheme 3:**
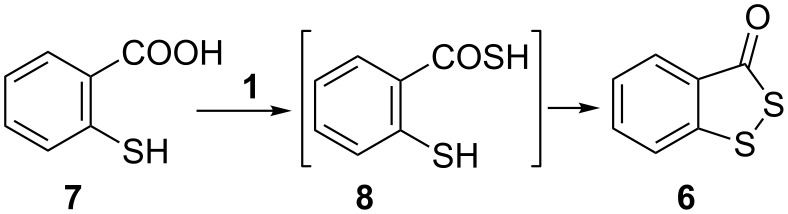
Synthesis of **6** by reaction between **7** and **1**.

**Scheme 4:**
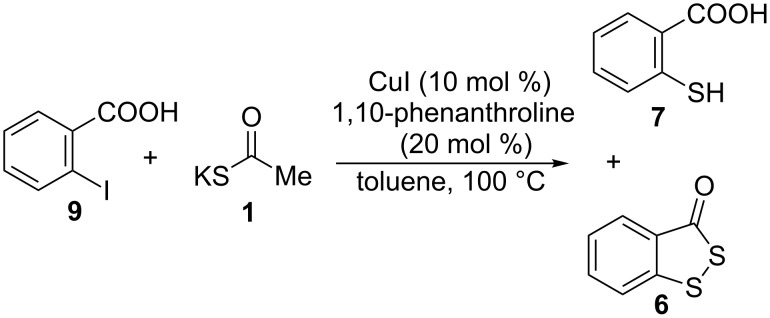
Reaction of 2-iodobenzoic acid (**9**) with **1**.

Afterwards we applied this procedure to the synthesis of heterocycles by the cross-coupling reaction of suitable *o*-substituted aryl iodides with KSCOMe. The reduced form of the Beaucage reagent **6**, a sulfurizing reagent used for the synthesis of phosphorothioate oligonucleotides [57], was synthesized by the reaction of 2-mercaptobenzoic acid (**7**) with KSCOMe (**1**), probably through the intermediate **8** (Scheme 3) [58]. We envisioned that compound **6** could be easily achieved by a cascade process starting with the copper-mediated reaction of 2-iodobenzoic acid (**9**) with salt **1** to yield the thioacetate precursor of **7** (Scheme 4).

When a toluene solution of **9** and **1** in a ratio of 1:1.5 was allowed to react in the presence of CuI/1,10-phenanthroline, a mixture of 2-mercaptobenzoic acid (**7**) and 3*H*-benzo[*c*][1,2]dithiol-3-one (**6**) was obtained in 10% and 27% isolated yields, respectively (Scheme 4).

**Scheme 5:**
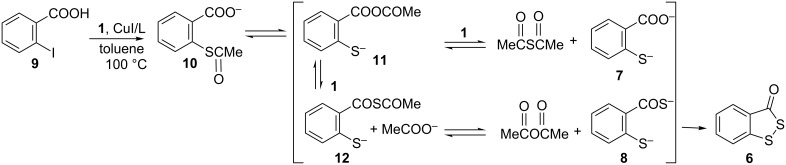
Suggested one-pot synthesis of heterocycle **6**.

Compounds **7** and **6** derive from the previous C–S coupling reaction. Thus, the expected 2-(acetylthio)benzoate (**10**) undergoes a series of acyl transfers, and therefore, thioanhydride **12**, and consecutively compound **8**, are formed. Irreversible oxidation of the latter finally affords heterocycle **6**. The free thiol **7** can be also generated by acyl transfer from 2-mercaptoanhydride **11** (Scheme 5).

By optimizing the reaction conditions, it was possible to improve the yield of **6**. Hence, the copper-catalyzed reaction between iodide **9** and **1** in a ratio of 1:2 rendered only heterocycle **6** (53% isolated yield) in a one-pot procedure. (Increasing the ratio from 1:2 to 1:3 did not increase the isolated yield of **6**). A possible mechanism to account for this involves a cascade of reactions starting with a cross-coupling and followed by consecutive acyl transfers (Scheme 5).

**Scheme 6:**
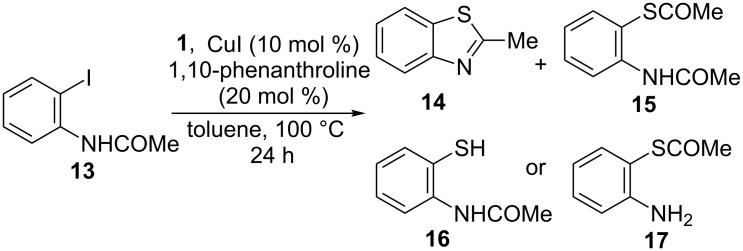
Reaction of *N*-(2-iodophenyl)acetamide (**13**) with **1**.

We also explored the possibility of building a benzothiazole ring by copper-catalyzed reaction of *N*-(2-iodophenyl)acetamide (**13**) with **1**. Thus, together with benzothiazole **14**, the reaction of **13** and **1** in a 1:1.5 ratio, in the presence of CuI/1,10-phenanthroline after 24 h of conventional heating, afforded low amounts of the substituted thioacetate derivative *S*-(2-acetoamidophenyl)thioacetate (**15**) and *N*-(2-mercaptophenyl)acetamide (**16**) or *S*-(2-aminophenyl)thioacetate (**17**) without cyclization, as detected by GC–MS spectrometry (Scheme 6).

**Scheme 7:**
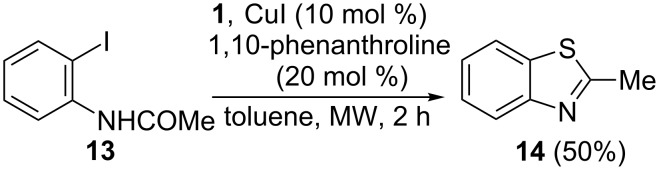
Synthesis of 2-methylbenzothiazole (**14**).

In order to accelerate the ring-closure reaction, a mixture of **13** and **1** in a 1:1.5 ratio was heated for 2 h under microwave irradiation, and 2-methylbenzothiazole (**14**) was isolated from the reaction mixture in 50% as the only reaction product (Scheme 7). Increasing the amount of **1** (**13**:**1** in a 1:3 ratio) does not significantly increase the yield of **14** (57%).

**Scheme 8:**
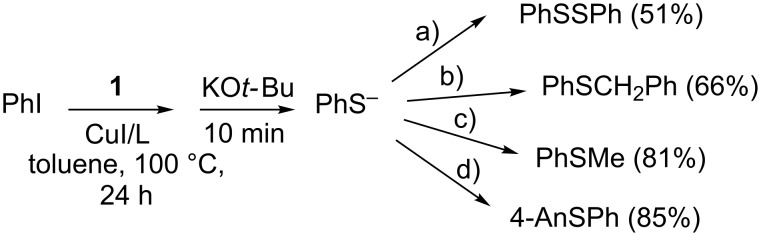
One-pot three-step synthesis of sulfides. (a) KI/I_2_; (b) BrCH_2_Ph; (c) MeI; (d) 4-AnI, CuI/1,10-phenanthroline (10 and 20 mol %, respectively), 100 °C, 24 h.

This methodology was finally applied to the one-pot three-step synthesis of alkyl aryl sulfides, diaryldisulfides and asymmetric diaryl sulfides. Thus, without isolation, the phenyl thioacetate produced in good yield by the copper-catalyzed reaction of PhI with thioacetate anion **1** was hydrolyzed to the benzene thiolate anion by the addition of 2 equiv of KO*t*-Bu. Different chemical transformations are possible from the arene thiolate ions formed; for example, oxidation to the diaryl disulfide, subsequent nucleophilic substitution reaction with alkyl halides to afford alkyl aryl sulfides, or a second copper-catalyzed reaction with a different aryl iodide to yield asymmetric diaryl sulfides. With this strategy we synthesized different sulfur compounds. Accordingly, a solution of benzene thiolate obtained from this methodology affords bis(phenyl)disulfide (51%) after oxidation by KI/I_2_, by subsequent substitution reactions with methyl iodide and benzyl bromide, methyl phenyl sulfide and benzyl methyl sulfide in 81% and 66% yields, respectively, and 4-anisyl phenyl sulfide (4-AnSPh) in 85% yield (quantified by ^1^H NMR) by a second copper-catalyzed reaction (Scheme 8).

## Conclusion

We have developed a simple, versatile, efficient and economically attractive procedure for the synthesis of sulfur heterocycles and a variety of sulfides with good yields. This one-pot methodology involves a cascade of reactions starting with a C–S bond formation by copper catalysis and followed by consecutive acyl transfers, condensation, nucleophilic substitution, oxidation or a second copper cross-coupling of the arene thiolate anion intermediates from *S*-aryl thioacetate hydrolysis. These thioacetates are obtained by copper-catalyzed reaction of commercial potassium thioacetate with aryl iodides under microwave irradiation. Electron-donor and electron-acceptor substituents are tolerated, and polysubstitution can also be successfully accomplished.

## Experimental

### Representative experimental procedures for the Cu-catalyzed base-free C–S coupling

Method A: The reactions were carried out in a 10 mL two-necked Schlenk tube, equipped with a nitrogen gas inlet, a condenser and a magnetic stirrer. The tube was dried under vacuum, filled with nitrogen and then charged with dried toluene (4.0 mL). ArI (0.5 mmol), CuI (10 mol %), 1,10-phenanthroline (20 mol %) and finally potassium thioacetate (**1**) (0.75 mmol) were added to the degassed solvent under nitrogen and stirred at 100 °C for 24 h. The reaction mixture was cooled to room temperature. Diethylether (20 mL) and water (20 mL) were added and the mixture was stirred. The organic layer was separated and the aqueous layer was extracted with diethylether (2 × 20 mL). The combined organic extract was dried over Na_2_SO_4_, and the products were isolated by radial chromatography from the crude reaction mixture or quantified by GC by using the internal standard method. The identity of all the products was confirmed by ^1^H and ^13^C NMR and MS spectrometry. All the thioacetate compounds prepared are known, and their data are in good agreement with those reported.

Method B: The reactions were carried out in a 10 mL glass tube, filled with nitrogen and then charged with dried toluene (2 mL). ArI (0.5 mmol), CuI (10 mol %), 1,10-phenanthroline (20 mol %) and finally potassium thioacetate (**1**) (0.75 mmol) were added to the degassed solvent under nitrogen. Then, the tube was sealed with a rubber cap and heated to 110–140 °C for 2 h under microwave irradiation (Fixed Power, 25 W) by using the SPS method. This method implies irradiation at 25 W to bring the reaction mixture to 140 °C, then cycling of the power on and off for the remaining run time (2 h) as the temperature varies from 110–140 °C. After completion of the experiment, the vessel was cooled to room temperature before removal from the microwave cavity, and then opened to the atmosphere. The work-up of the reaction was similar to that of Method A.

### Representative procedure for the one-pot three-step synthesis of alkyl aryl sulfides, diaryl disulfides and asymmetric diaryl sulfides

The reactions were carried out by using method A. After 24 h at 100 °C, KO*t*-Bu (1 mmol, 2 equiv) was added to the reaction mixture and stirred for 10 min. The corresponding alkyl halide (0.75 mmol, 1.5 equiv) or KI/I_2_ (1.5 mmol/0.51 mmol, 3/1.02 equiv) was then added and stirred for 20 min or 24 h, respectively. The work-up of the reactions was similar to that of Method A.

For the synthesis of the asymmetric diaryl sulfide, after hydrolysis of the thioester, a second addition of CuI/1,10-phenanthroline (10 and 20 mol %, respectively) was required, together with the new aryl iodide (1 equiv). After stirring for 24 h at 100 °C, the work-up of the reaction was similar to that of Method A.

## Supporting Information

File 1 Experimental details**,** characterization data and spectra (^1^H, ^13^C NMR, and HSQC or HMBC as consigned) for all the products (**2a–i**, **3–7**, and **14**).
