# Supplementary material for: Efficient Cu-catalyzed base-free C–S coupling under conventional and microwave heating. A simple access to S-heterocycles and sulfides
Source: Beilstein J Org Chem. 2013 Mar 4;9:467–75. doi: 10.3762/bjoc.9.50 (PMC3596051; doi:10.3762/bjoc.9.50)

Supporting Information

for

Efficient Cu-catalyzed base-free C–S coupling under conventional and microwave heating. A simple access to S-heterocycles and sulfides

Silvia M. Soria-Castro and Alicia B. Peñéñory*

Address: INFIQC, Departamento de Química Orgánica, Facultad de Ciencias Químicas, Universidad Nacional de Córdoba, Ciudad Universitaria, X5000HUA. Córdoba, Argentina, Fax: (+54) 351-4333030.

Email: Alicia B. Peñéñory - penenory@fcq.unc.edu.ar

* Corresponding author

Experimental details, characterization data and spectra

(^{1}H, ^{13}C NMR, and HSQC or HMBC as consigned)

for all the products (2a–i, 3–7, and 14)

Experimental section

Materials and methods ……..…………………..…………..……..……….………………......….. S3

Representative experimental procedures for the Cu-catalyzed base-free C–S coupling ………..… S4

Representative procedure for the one-pot three-step synthesis of alkyl aryl sulfides, diaryl disulfides and asymmetric diaryl sulfides ………………..….. S5

Characterization data of compounds

S-Phenyl thioacetate (**2a**), *S*-*p*-tolyl thioacetate (**2b**), *S*-(4-methoxyphenyl) thioacetate (**2c**), *S*-(4-acetamidophenyl) thioacetate (**2d**), *S*-(4-acetylphenyl) thioacetate (**2e**), *S*-(4-nitrophenyl) thioacetate (**2f**), *S*-(4-cyanophenyl) thioacetate (**2g**), *S*-*o*-tolyl thioacetate (**2h**), *S*-(2-methoxyphenyl) thioacetate (**2i**), *S*-pyridin-3-yl thioacetate (**3**), *S*-naphthalen-1-yl thioacetate (**4**), *S,S'*-1,4-phenylene dithioacetate (**5**), 3*H*-benzo[*c*][1,2]dithiol-3-one (**6**), 2-mercaptobenzoic acid (**7**) and 2-methylbenzothiazole (**14**) ……………………………………………………………..…… S6–S9

References ……………………………………………………...………………..………..… S9

^{1}H NMR and ^{13}C NMR spectra, and HSQC or HMBC as consigned of compounds **2a–i**, **3–7** and **14** …..…………………………………………………………………………………….... S10–S34

Experimental section

Materials and methods

Chemicals: KSCOSMe, KO*t*-Bu, aryl iodides, alkyl halides, 1,10-phenanthroline, L-proline, tetramethylethylenediamine (TMEDA), dimethylethylenediamine (DMEDA), benzotriazole and acetylacetone were all high-purity, commercially available reagents used without further purification. The commercially available copper salts CuI (>98%), CuCl (90%), $CuCl_2 \cdot H_2O$ (reagent grade), $Cu(OAc)_2$ (98%), $Cu(OTf)_2$ (98%), CuO (99%) were used as received. DMSO absolute grade was used without further purification and stored over molecular sieves (4 Å). Toluene was distilled by standard procedures and stored over molecular sieves (4 Å). All the reaction products were isolated by radial chromatography (silica gel, petroleum/ethyl ether) from the reaction mixture and characterized by ^1H and ^{13}C NMR and mass spectrometry. All the *S*-aryl thioacetates synthesized are known; they exhibited physical properties identical to those reported in the literature.

General methods: ^1H and ^{13}C NMR spectra were recorded at 400.16 and 100.62 MHz, respectively, on a Bruker Advance II 400 spectrometer, and all spectra were reported in δ (ppm) relative to Me_4Si, with $CDCl_3$ as solvent. Gas chromatographic analyses were performed with a flame-ionization detector, on 30 m capillary column of a 0.32 mm × 0.25 μm film, with a 5% phenylpolysiloxane phase. GS–MS analyses were performed employing a 25 m × 0.2 mm × 0.33 μm column with a 5% phenylpolysiloxane phase. Ionization was achieved by electronic impact (70 eV) and detection set up in positive mode. Microwave-induced reactions were performed in a single-mode instrument (CEM Focused Microwave TM Synthesis System, Model Discover) equipped with a noncontact infrared

sensor to measure the temperature, a direct pressure control system to measure the pressure of the reaction vessel content, and cooling system employing compressed air.

Representative experimental procedures for the Cu-catalyzed base-free C–S coupling:

Method A: The reactions were carried out in a 10 mL two-necked Schlenk tube, equipped with a nitrogen gas inlet, a condenser and a magnetic stirrer. The tube was dried under vacuum, filled with nitrogen and then charged with dried toluene (4.0 mL). ArI (0.5 mmol), CuI (10 mol %), 1,10-phenanthroline (20 mol %) and finally potassium thioacetate (**1**) (0.75 mmol) were added to the degassed solvent under nitrogen and stirred at 100 °C for 24 h. The reaction mixture was cooled to room temperature. Diethylether (20 mL) and water (20 mL) were added, and the mixture was stirred. The organic layer was separated, and the aqueous layer was extracted with diethylether (2 × 20 mL). The combined organic extract was dried over Na_2SO_4 (anh), and the products were isolated by radial chromatography from the crude reaction mixture or quantified by GC using the internal standard method. The identity of all the products was confirmed by ^1H and ^{13}C NMR and MS spectrometry. All the thioacetate compounds prepared are known, and their data are in good agreement with those reported.

Method B: The reactions were carried out in a 10 mL glass tube, filled with nitrogen and then charged with dried toluene (2 mL). ArI (0.5 mmol), CuI (10 mol %), 1,10-phenanthroline (20 mol %) and finally potassium thioacetate (**1**) (0.75 mmol) were added to the degassed solvent under nitrogen. Then, the tube was sealed with a rubber cap and heated to 110–140 °C for 2 h under microwave irradiation (Fixed Power, 25 W) using the SPS method. This method implies irradiation at 25 W to bring the reaction mixture to 140 °C, then cycling of the power on and off for the remaining run time (2 h) as the

temperature varies from 110 °C to 140 °C. After completion of the experiment, the vessel was cooled to room temperature before removal from the microwave cavity, and then opened to the atmosphere. The work-up of the reaction was similar to that of Method A.

Representative procedure for the one-pot three-step synthesis of alkyl aryl sulfides, diaryl disulfides and asymmetric diaryl sulfides:

The reactions were carried out by using method A. After 24 h at 100 °C, KO*t*-Bu (1 mmol, 2 equiv) was added to the reaction mixture and stirred for 10 min. The corresponding alkyl halide (0.75 mmol, 1.5 equiv) or KI/I_2 (1.5 mmol/0.51 mmol, 3/1.02 equiv) was then added and stirred for 20 min or 24 h, respectively. The work-up of the reactions was similar to that of Method A.

For the synthesis of the asymmetric diaryl sulfide, after hydrolysis of the thioester, a second addition of CuI/1,10-phenanthroline (10 and 20 mol %, respectively) was required, together with the new aryl iodide (1 equiv). After stirring for 24 h at 100 °C, the work-up of the reaction was similar to that of Method A.

Synthesis of 3*H*-benzo[*c*][1,2]dithiol-3-one (6): The product was obtained by the reaction between 2-iodobenzoic acid (**9**) and **1** in a ratio of 1:2 by the method A. The reaction product was isolated by column chromatography (silica gel, petroleum/ethyl ether, 95/5) in 53% yield.

Synthesis of 2-methylbenzothiazole (14): The product was obtained by the reaction between *N*-(2-iodophenyl)acetamide (**13**) and **1** in a ratio of 1:1.5 by method B. The reaction product was isolated by column chromatography (silica gel, petroleum/ethyl ether, 95/5) in 50% yield.

Characterization data of compounds

***S*-Phenyl thioacetate (2a)** [1]: ^{1}H NMR (400 MHz, $CDCl_3$) δ 2.42 (s, 3H), 7.41 (s, 5H). ^{13}C NMR (101 MHz, $CDCl_3$) δ 30.2, 127.9, 129.2, 129.5, 134.5, 194.1; GC-MS (*m*/*z*): 152 (M^+, 14), 111 (8), 110 (100), 109 (18), 84 (6), 69 (8), 66 (14), 65 (15), 51 (6), 50 (4).

***S*-*p*-Tolyl thioacetate (2b)** [1]: ^{1}H NMR (400 MHz, $CDCl_3$) δ 2.37 (s, 3H), 2.40 (s, 3H), 7.22 (d, *J* = 8.0 Hz, 2H), 7.29 (d, *J* = 8.2 Hz, 2H); ^{13}C NMR (101 MHz, $CDCl_3$) δ 21.3, 30.1, 124.5, 130.1, 134.4, 139.7, 194.6; ^{1}H/^{13}C HSQC NMR ($CDCl_3$) δ_H/δ_C δ 2.37/21.3, 2.40/30.1, 7.22/130.1, 7.29/134.4.

***S*-(4-Methoxyphenyl) thioacetate (2c)** [2]: ^{1}H NMR (400 MHz, $CDCl_3$) δ 2.49 (s, 3H), 3.92 (s, 3H), 7.04 (d, *J* = 8.9 Hz, 2H), 7.42 (d, *J* = 8.9 Hz, 2H); ^{13}C NMR (101 MHz, $CDCl_3$) δ 29.9, 55.4, 114.9, 118.7, 136.1, 160.7, 195.2; ^{1}H/^{13}C HSQC NMR ($CDCl_3$) δ_H/δ_C δ 2.49/29.9, 3.92/55.4, 7.04/114.9, 7.42/136.1.

***S*-(4-Acetamidophenyl) thioacetate (2d)** [3]: ^{1}H NMR (400 MHz, $CDCl_3$) δ 2.11 (s, 3H), 2.42 (s, 3H), 7.30 (d, *J* = 8.5 Hz, 1H), 7.51 (d, *J* = 8.5 Hz, 1H), 7.92 (s, br, 1H); ^{13}C NMR (101 MHz, $CDCl_3$) δ 24.5, 30.1, 120.3, 122.2, 135.3, 139.5, 168.8, 195.6; ^{1}H/^{13}C HSQC NMR ($CDCl_3$) δ_H/δ_C δ 2.11/24.5, 2.42/30.1, 7.30/135.3, 7.51/120.3; ^{1}H/^{13}C HMBC NMR ($CDCl_3$) δ_H/δ_C δ 2.11/168.8, 2.42/195.6, 7.30/120.3, 7.30/139.5, 7.51/122.2, 7.55/135.3.

***S*-(4-Acetylphenyl) thioacetate (2e)** [4]: ^{1}H NMR (400 MHz, $CDCl_3$) δ 2.45 (s, 3H), 2.61 (s, 3H), 7.52 (d, *J* = 8.4 Hz, 2H), 7.97 (d, *J* = 8.4 Hz, 2H); ^{13}C NMR (101 MHz, $CDCl_3$) δ 26.7, 30.5, 128.8, 133.8, 134.2, 137.4, 192.6, 197.3; ^{1}H/^{13}C HSQC NMR ($CDCl_3$) δ_H/δ_C δ 2.45/30.5, 2.61/26.7, 7.52/134.2, 7.97/128.8; ^{1}H/^{13}C HMBC NMR ($CDCl_3$) δ_H/δ_C δ 2.45/192.6, 2.61/197.3, 7.52/137.4, 7.97/133.8, 7.97/197.3.

***S*-(4-Nitrophenyl) thioacetate (2f)** [5]: ^{1}H NMR (400 MHz, $CDCl_3$) δ 2.49 (s, 3H), 7.60 (d, *J* = 8.7 Hz, 2H), 8.25 (d, *J* = 8.7 Hz, 2H); ^{13}C NMR (101 MHz, $CDCl_3$) δ 30.6, 124.0,

134.6, 136.3, 148.2, 191.6; $^1H/^{13}C$ HSQC NMR ($CDCl_3$) δ_H/δ_C δ 2.49/30.6, 7.60/134.6, 8.25/124.0; $^1H/^{13}C$ HMBC NMR ($CDCl_3$) δ_H/δ_C δ 2.49/191.6, 7.60/124.0, 7.60/148.2, 8.25/136.3, 8.25/148.2; GC–MS (*m/z*): 197 (M^+, 45), 167 (5), 155 (31), 154 (7), 140 (5), 139 (44), 138 (7), 126 (4), 125 (56), 124 (14), 110 (8), 109 (55), 108 (42), 97 (20), 96 (10), 83 (8), 82 (51), 81 (17), 80 (8), 76 (9), 75 (13), 74 (15), 70 (21), 69 (100), 65 (44), 64 (12), 63 (56), 62 (14), 58 (24), 51 (16), 50 (29).

***S*-(4-Cyanophenyl) thioacetate (2g)** [6]: ^1H NMR (400 MHz, $CDCl_3$) δ 2.47 (s, 3H), 7.53 (d, *J* = 8.6 Hz, 1H), 7.68 (d, *J* = 8.6 Hz, 1H); ^{13}C NMR (101 MHz, $CDCl_3$) δ 30.5, 113.0, 118.2, 132.5, 134.2, 134.6, 191.8; $^1H/^{13}C$ HSQC NMR ($CDCl_3$) δ_H/δ_C δ 2.47/30.5, 7.53/134.6, 7.68/132.5; $^1H/^{13}C$ HMBC NMR ($CDCl_3$) δ_H/δ_C δ 2.47/191.8, 7.53/113.0, 7.68/118.2, 7.68/134.2.

***S*-*o*-Tolyl thioacetate (2h)** [7]: ^1H NMR (400 MHz, $CDCl_3$) δ 2.34 (s, 3H), 2.40 (s, 3H), 7.18–7.23 (m, 1H), 7.29–7.32 (m, 2H), 7.38 (d, *J* = 7.5 Hz, 1H); ^{13}C NMR (101 MHz, $CDCl_3$) δ 20.8, 30.2, 126.7, 127.5, 130.2, 130.8, 135.9, 142.0, 193; $^1H/^{13}C$ HSQC NMR ($CDCl_3$) δ_H/δ_C δ 2.34/20.8, 2.40/30.2, 7.18–7.23/126.7, 7.29–7.32/130.2, 7.29–7.32/130.8, 7.38/135.9.

***S*-(2-Methoxyphenyl) thioacetate (2i)** [8]: ^1H NMR (400 MHz, $CDCl_3$) δ 2.40 (s, 3H), 3.84 (s, 3H), 6.97–7.00 (m, 2H), 7.45–7.35 (m, 2H); ^{13}C NMR (101 MHz, $CDCl_3$) δ 29.8, 55.8, 111.5, 115.9, 121.0, 131.7, 136.6, 159.1, 194.2; $^1H/^{13}C$ HSQC NMR ($CDCl_3$) δ_H/δ_C δ 2.40/29.8, 3.84/55.8, 6.97–7.00/111.5, 6.97–7.00/121.0, 7.45–7.35/131.7, 7.45–7.35/136.6.

***S*-Pyridin-3-yl thioacetate (3)** [9]: ^1H NMR (400 MHz, $CDCl_3$) δ 2.46 (s, 3H), 7.36 (dd, *J* = 7.9, 4.9 Hz, 1H), 7.71–7.76 (m, 1H), 8.57 (d, *J* = 1.7 Hz, 1H), 8.62 (dd, *J* = 4.8, 1.4 Hz, 1H); ^{13}C NMR (101 MHz, $CDCl_3$) δ 30.4, 124.0, 125.4, 142.0, 150.2, 154.0, 192.5; $^1H/^{13}C$ HSQC NMR ($CDCl_3$) δ_H/δ_C δ 2.46/30.4, 7.36/124.0, 7.71–7.76/142.0, 8.57/154.0,

8.62/150.2; ^{1}H\$^{13}$C HMBC NMR ($CDCl_3$) δ_H/δ_C δ 2.46/192.5, 7.36/125.4, 7.36/150.2, 7.71–7.76/150.2, 7.71–7.76/154.0, 8.57/125.4, 8.57/142.0, 8.57/150.2, 8.62/124.0, 8.62/142.0, 8.62/154.0.

***S*-Naphthalen-1-yl thioacetate (4)** [10]: ^{1}H NMR (400 MHz, $CDCl_3$) δ 2.44 (s, 3H), 7.44–7.58 (m, 3H), 7.68 (d, *J* = 7.1 Hz, 1H), 7.86 (d, *J* = 7.6 Hz, 1H), 7.92 (d, *J* = 8.3 Hz, 1H), 8.18 (d, *J* = 8.2 Hz, 1H); ^{13}C NMR (101 MHz, $CDCl_3$) δ 30.3, 125.3, 125.5, 125.6, 126.5, 127.3, 128.7, 131.0, 134.2, 135.0, 194.2; ^{1}H/^{13}C HSQC NMR ($CDCl_3$) δ_H/δ_C δ 2.44/30.3, 7.44–7.58/125.6, 7.44–7.58/126.5, 7.44–7.58/127.3, 7.68/135.0, 7.86/128.7, 7.92/135.0, 8.18/125.3; GC–MS (*m*/*z*): 203 (M^+ + 1, 1), 202 (M^+, 14), 162 (6), 161 (12), 160 (100), 159 (10), 158 (3), 129 (2), 128 (10), 127 (2), 126 (4), 116 (8), 115 (38), 114 (5), 89 (4), 88 (2), 79 (2), 75 (2), 69 (3), 63 (3).

***S*,*S*'-1,4-Phenylene dithioacetate (5)** [11]: ^{1}H NMR (400 MHz, $CDCl_3$) δ 2.43 (s, 6H), 7.45 (s, 4H); ^{13}C NMR (101 MHz, $CDCl_3$) δ 30.3, 129.6, 134.8, 193.1; GC–MS (*m*/*z*): 228 (1), 226 (M^+, 7), 186 (3), 185 (5), 184 (40), 144 (5), 143 (5), 142 (54), 141 (9), 108 (4), 97 (4), 78 (10), 69 (6), 43 (100).

3*H*-Benzo[*c*][1,2]dithiol-3-one (6) [12]: ^{1}H NMR (400 MHz, $CDCl_3$) δ 7.41 (ddd, *J* = 7.9, 6.2, 1.7 Hz, 1H), 7.68–7.60 (m, 2H), 7.96 (d, *J* = 7.9 Hz, 1H); ^{13}C NMR (101 MHz, $CDCl_3$) δ 124.7, 125.7, 127.4, 129.2, 133.5, 148.3, 193.7; ^{1}H/^{13}C HSQC NMR ($CDCl_3$) δ_H/δ_C δ 7.41/125.7, 7.68–7.60/124.7, 7.68–7.60/133.5, 7.96/127.4.

2-Mercaptobenzoic acid (7) [13]: ^{1}H NMR (400 MHz, $CDCl_3$) δ 6.49 (s, 1H), 7.22–7.37 (m, 4H); ^{13}C NMR (101 MHz, $CDCl_3$) δ 125.4, 126.6, 131.1, 131.3, 135.7, 142.6, 197.4.

2-Methylbenzothiazole (14): ^{1}H NMR (400 MHz, $CDCl_3$) δ 2.84 (s, 3H), 7.35 (t, *J* = 8 Hz, 1H), 7.45 (d, *J* = 8 Hz, 1H), 7.83 (d, *J* = 8 Hz, 1H), 7.95 (d, *J* = 8 Hz, 1H); ^{13}C NMR (101 MHz, CDCl3) δ 20.1, 121.4, 122.4, 124.7, 125.9, 135.7, 153.4, 167.0; ^{1}H/^{1}H COSY

NMR ($CDCl_3$) δH/δH δ 7.35/7.45, 7.35/7.83, 7.45/7.95; $^{1}H/^{13}C$ HSQC NMR ($CDCl_3$) δH/δC δ 2.84/20.1, 7.35/124.7, 7.45/125.9, 7.83/121.4, 7.95/122.4; $^{1}H/^{13}C$ HMBC NMR ($CDCl_3$) δH/δC δ 2.84/167.0, 7.35/122.4, 7.35/135.7, 7.45/121.4, 7.45/153.4, 7.83/125.9, 7.83/153.4, 7.95/124.7, 7.95/135.7.

References

1. Qiu, R. H.; Xu, X. H.; Li, Y. H.; Shao, L. L.; Zhang, G. P.; An, D. L. *Synth. Commun.* **2010,** *40*, 3309–3314. doi:10.1080/00397910903419803
2. Lubkoll, J.; Wennemers, H. *Angew. Chem., Int. Ed.* **2007,** *46*, 6841–6844. doi:10.1002/anie.200702187
3. Collings, A. J.; Morgan, K. J. *Tetrahedron* **1964,** *20*, 2167–2175. doi:10.1016/S0040-4020(01)97600-8
4. Overberger, C. G.; Lebovits, A. *J. Am. Chem. Soc.* **1956,** *78*, 4792–4797. doi:10.1021/ja01599a067
5. Dan, W.; Deng, H.; Chen, J.; Liu, M.; Ding, J.; Wu, H. *Tetrahedron* **2010,** *66*, 7384–7388. doi:10.1016/j.tet.2010.07.023
6. Barbero, M.; Degani, I.; Dughera, S.; Fochi, R. *Synthesis* **2003,** 1225–1230. doi:10.1055/s-2003-39413
7. Liu, R.; Li, Y.-H.; Chang, J.; Xiao, Q.; Zhu, H.-J. *Asian J. Chem.* **2010,** *22*, 3059–3064.
8. Zhou, G.; Yost, J. M.; Coltart, D. M. *Synthesis* **2007,** 478–482. doi:10.1055/s-2006-958959
9. Lai, C.; Backes, B. J. *Tetrahedron Lett.* **2007,** *48*, 3033–3037. doi:10.1016/j.tetlet.2007.02.128
10. Petrillo, G.; Novi, M.; Garbarino, G.; Filiberti, M. *Tetrahedron Lett.* **1989,** *45*, 7411–7420. doi:10.1016/S0040-4020(01)89203-6
11. Kim, B. S.; Choi, S. H.; Zhu, X.-Y.; Frisbie, C. D. *J. Am. Chem. Soc.* **2011,** *133*, 19864–19877. doi:10.1021/ja207751w
12. Palazzo, S.; Giannola, L. I.; Neri, M. *J. Heterocycl. Chem.* **1975,** *12*, 1077–1078. doi:10.1002/jhet.5570120560
13. Xu, H.-J.; Liang, Y.-F.; Cai, Z.-Y.; Qi, H.-X.; Yang, C.-Y.; Feng, Y.-S. *J. Org. Chem.* **2011,** *76*, 2296–2300. doi:10.1021/jo102506x

^{1}H NMR (400 MHz, $CDCl_3$) *S*-phenyl thioacetate (2a)

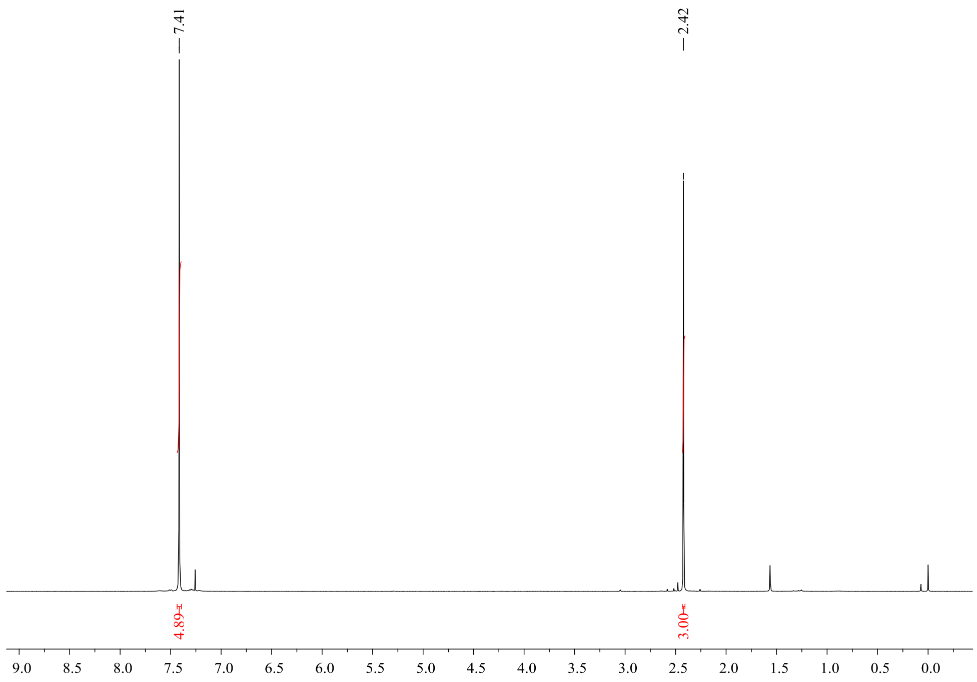

^{13}C NMR (101 MHz, $CDCl_3$) *S*-phenyl thioacetate (2a)

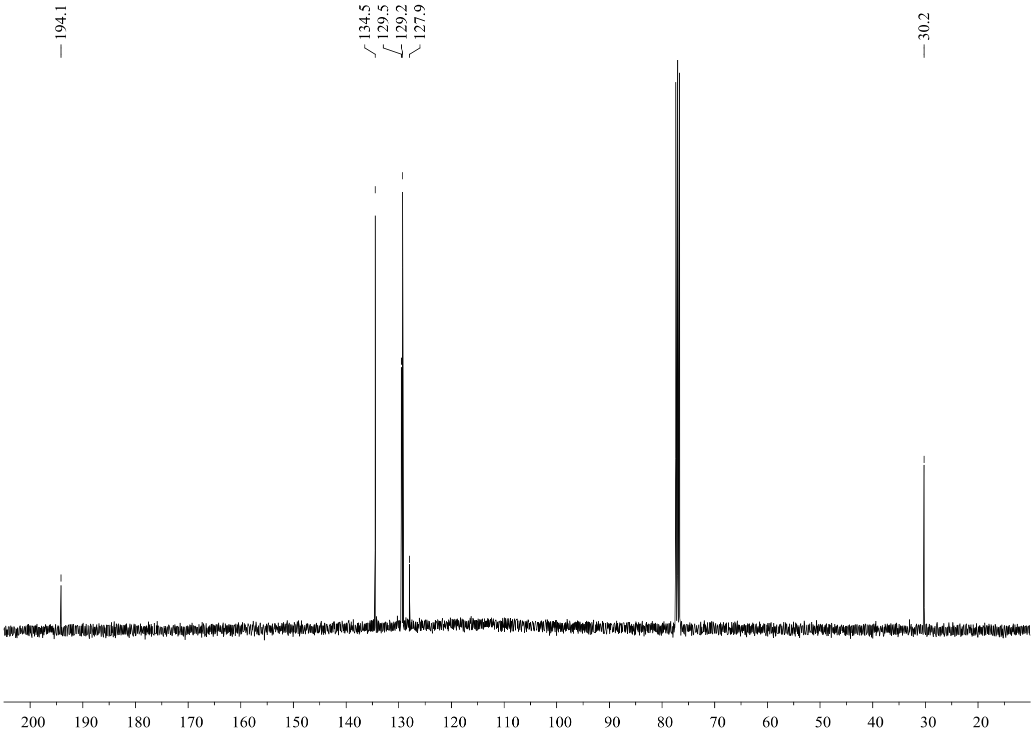

^{1}H NMR (400 MHz, $CDCl_3$) *S*-*p*-tolyl thioacetate (2b)

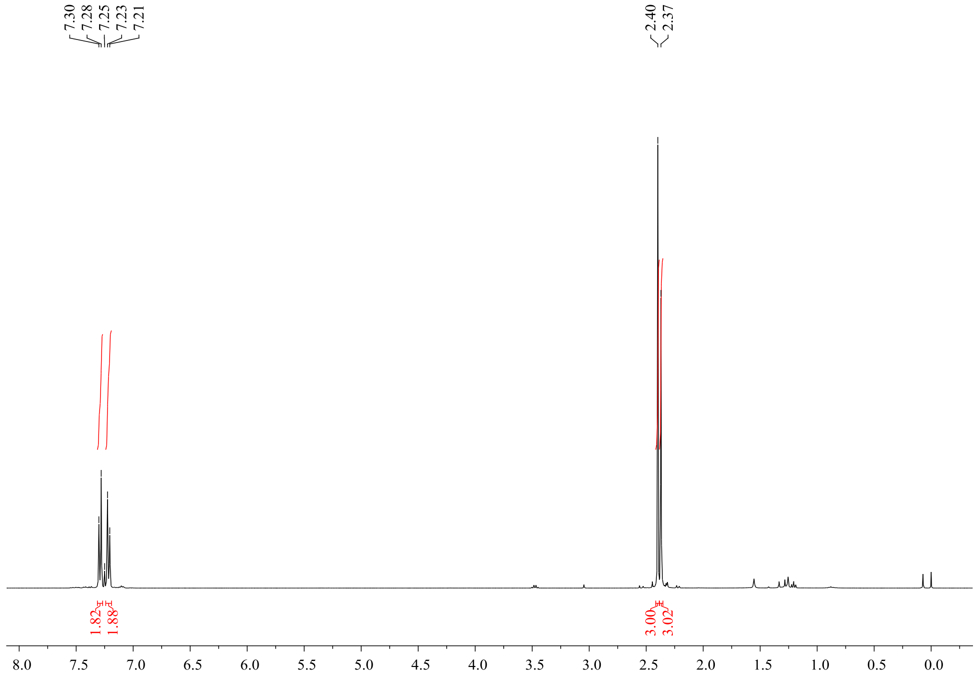

^{13}C NMR (101 MHz, $CDCl_3$) *S*-*p*-tolyl thioacetate (2b)

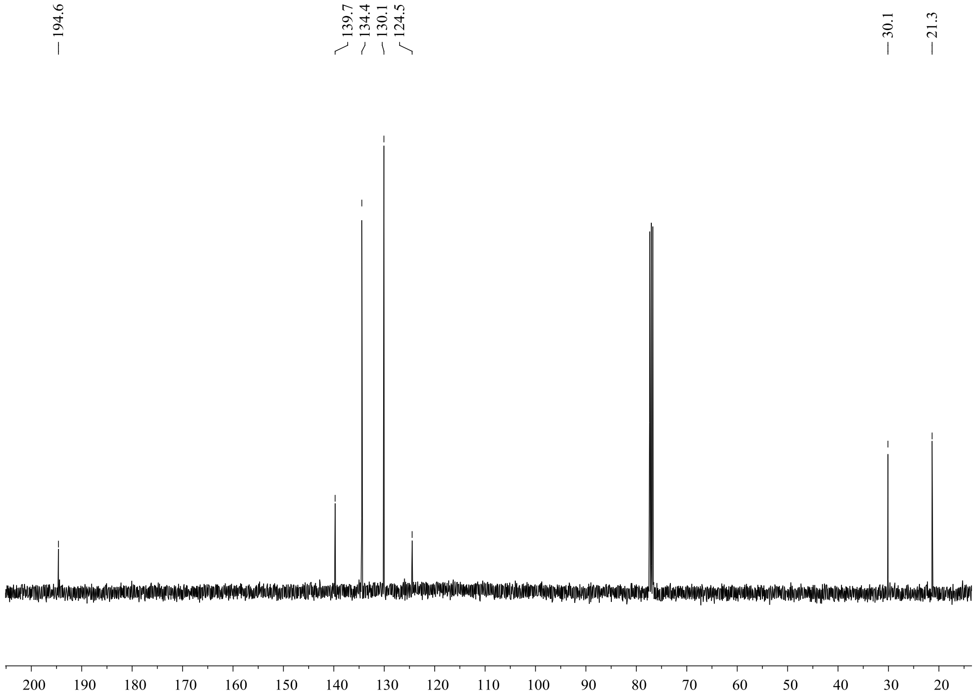

$^{1}H/^{13}C$ HSQC NMR ($CDCl_3$) *S*-*p*-tolyl thioacetate (2b)

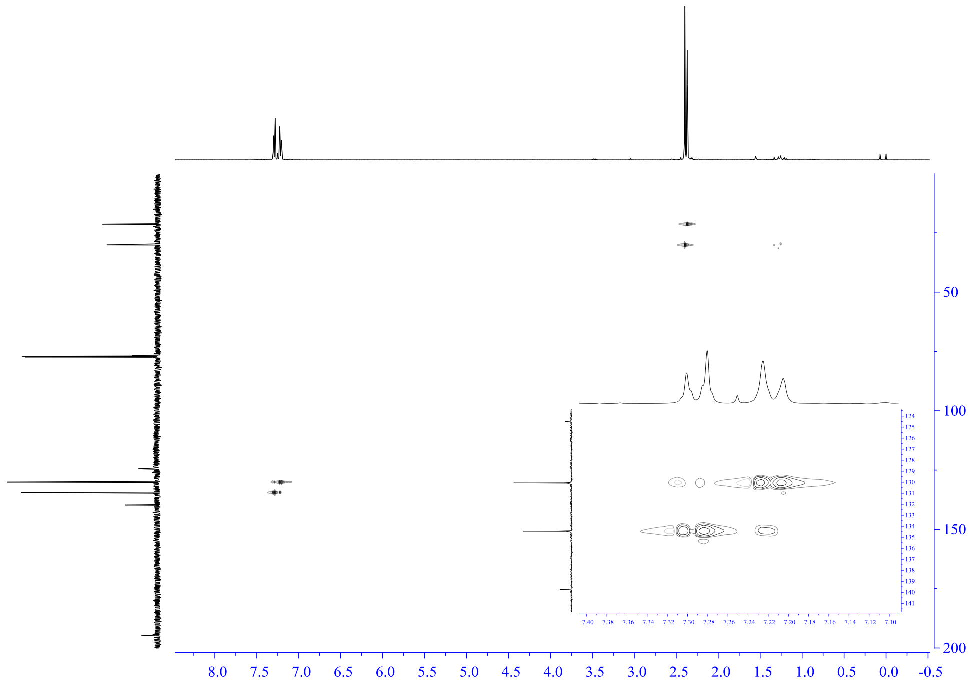

^{1}H NMR (400 MHz, $CDCl_3$) *S*-(4-methoxyphenyl) thioacetate (2c)

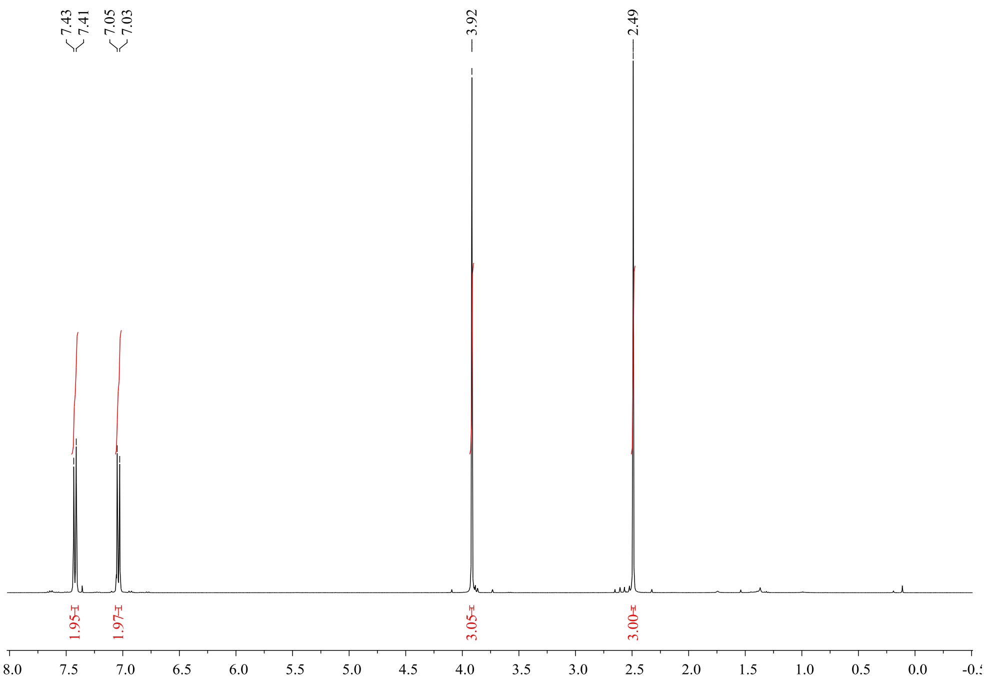

^{13}C NMR (101 MHz, $CDCl_3$) *S*-(4-methoxyphenyl) thioacetate (2c)

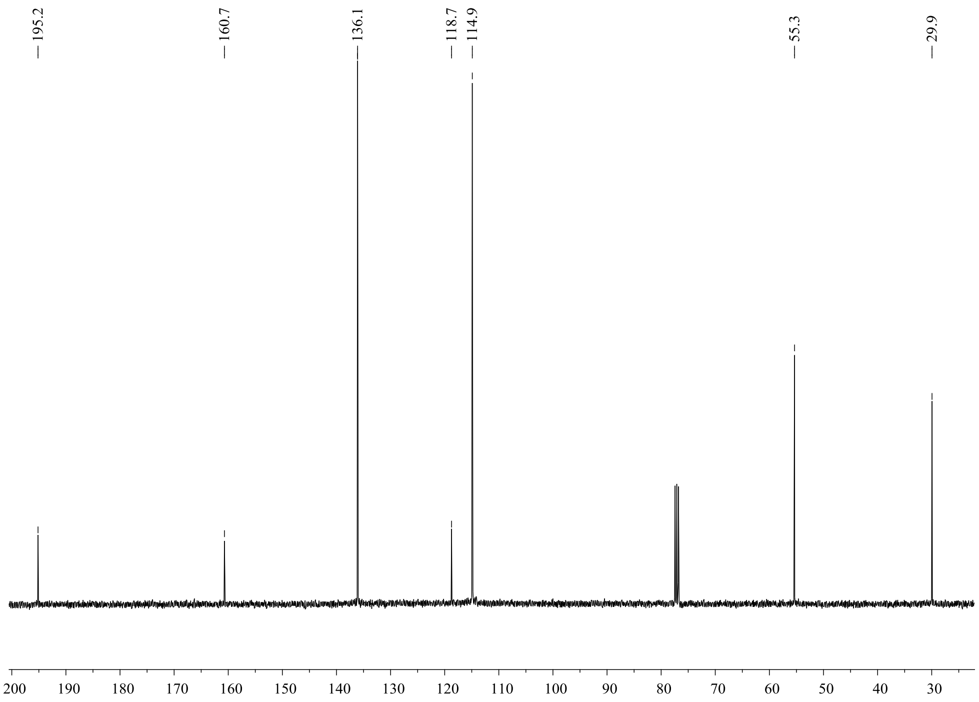

^{1}H/^{13}C HSQC NMR ($CDCl_3$) *S*-(4-methoxyphenyl) thioacetate (2c)

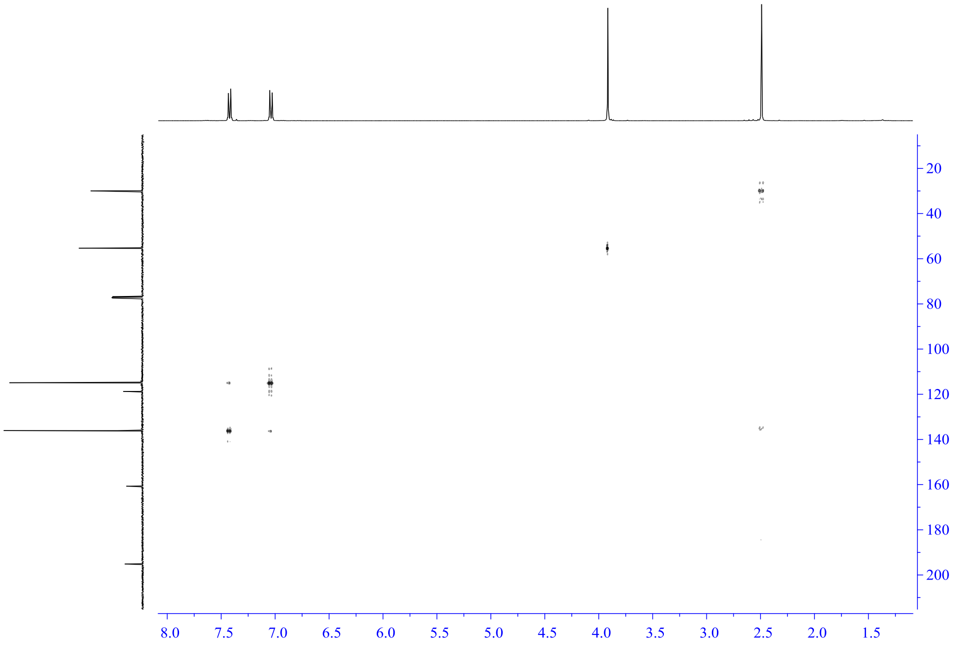

^{1}H NMR (400 MHz, $CDCl_3$) *S*-(4-acetamidophenyl) thioacetate (2d)

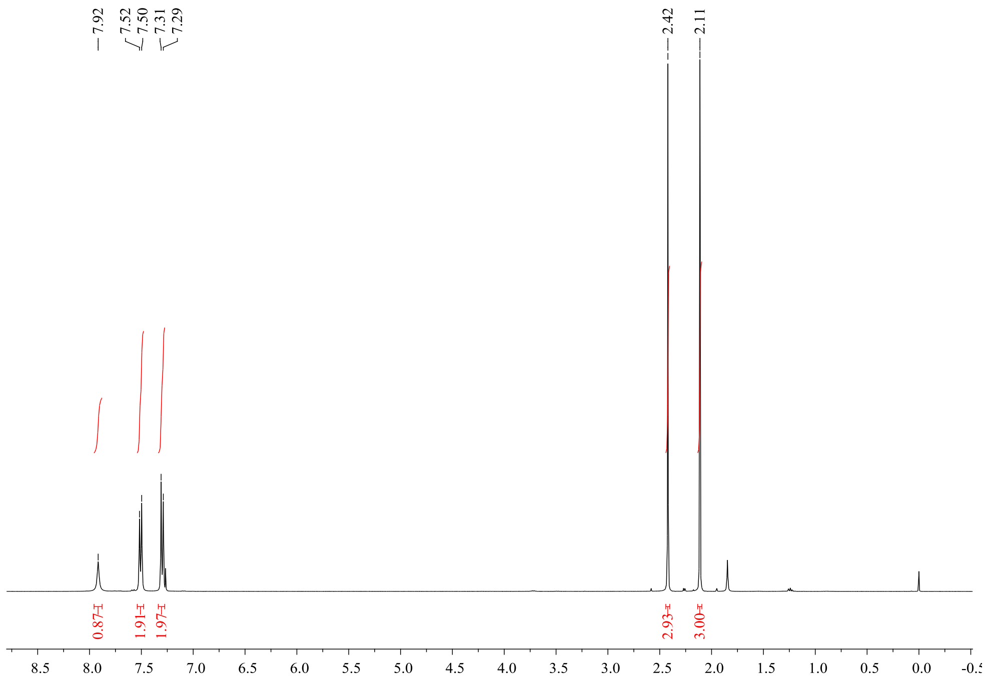

^{13}C NMR (101 MHz, $CDCl_3$) *S*-(4-acetamidophenyl) thioacetate (2d)

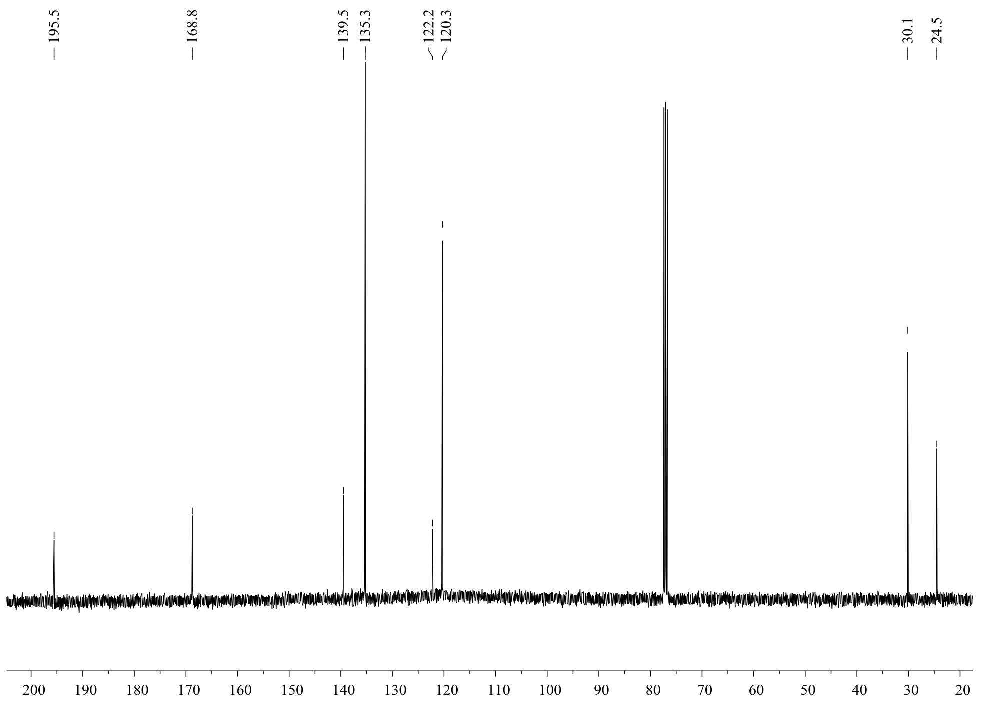

$^{1}H/^{13}C$ HSQC NMR ($CDCl_3$) *S*-(4-acetamidophenyl) thioacetate (2d)

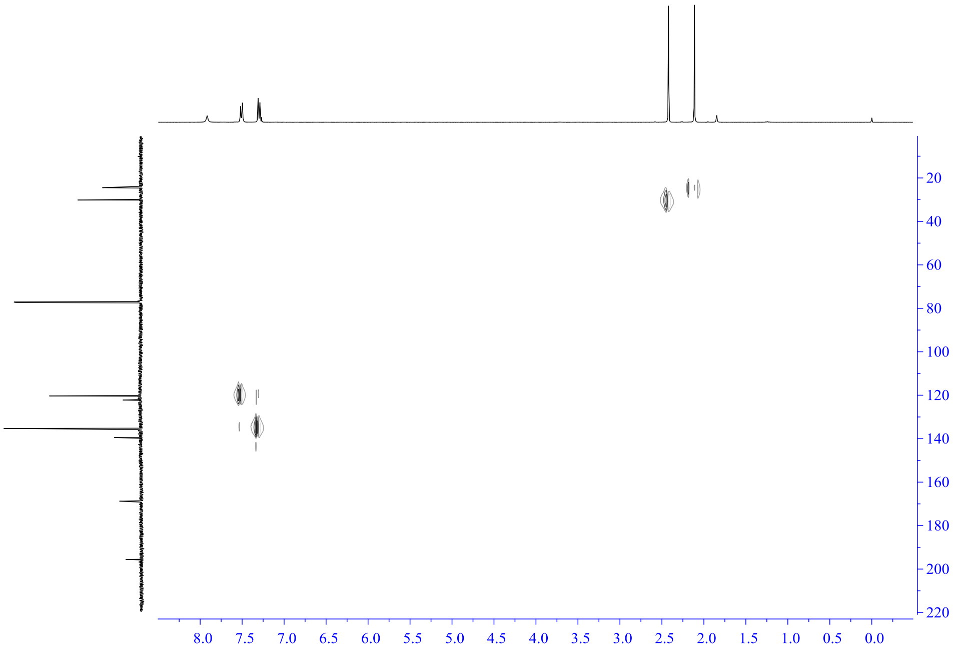

$^{1}H/^{13}C$ HMBC NMR ($CDCl_3$) *S*-(4-acetamidophenyl) thioacetate (2d)

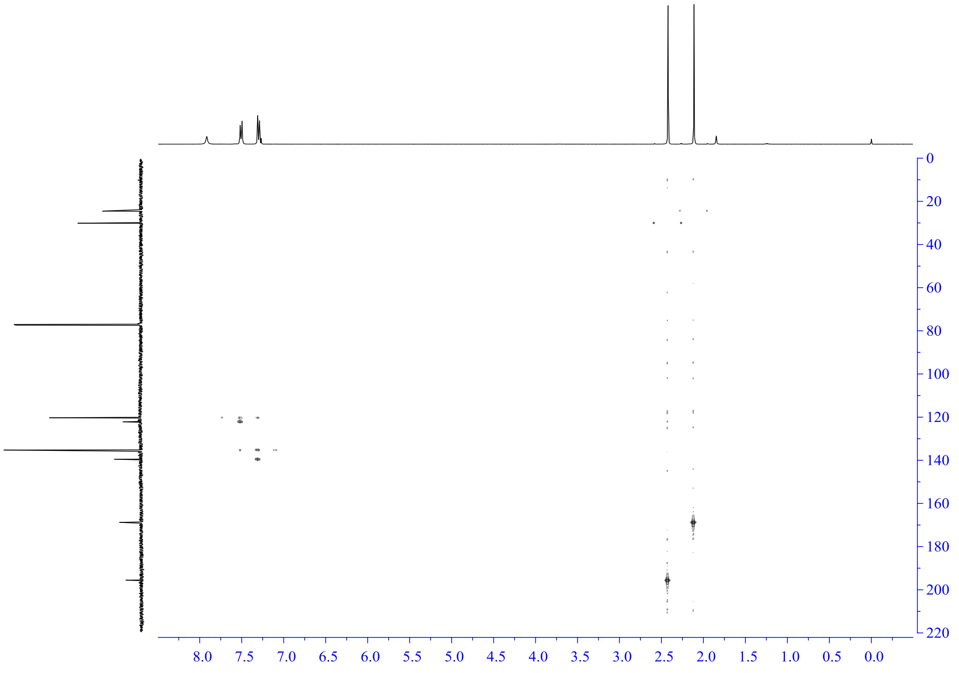

^{1}H NMR (400 MHz, $CDCl_3$) *S*-(4-acetylphenyl) thioacetate (2e)

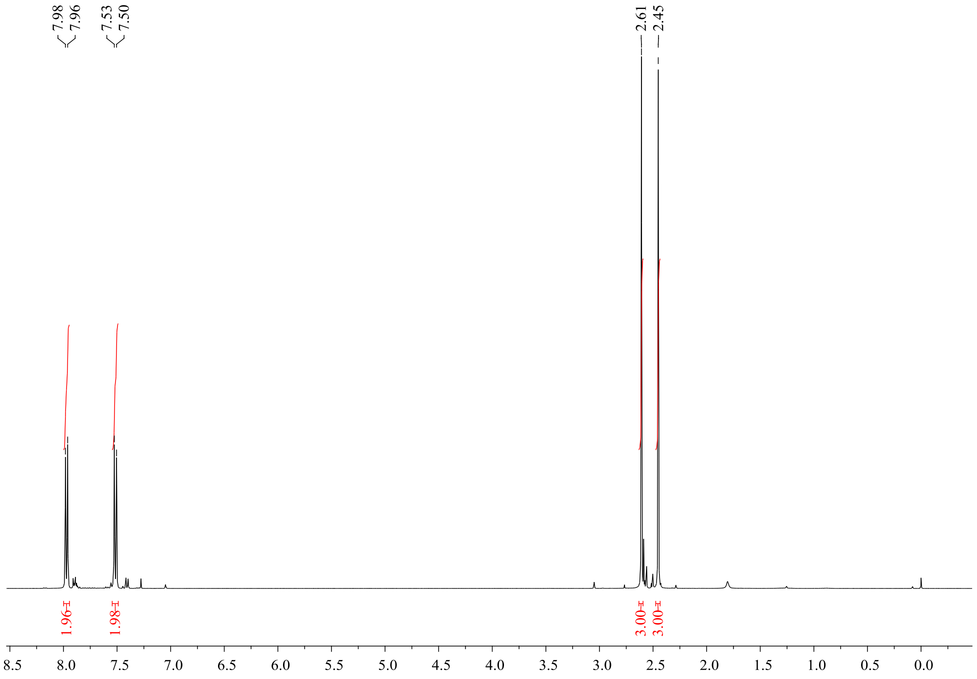

^{13}C NMR (101 MHz, $CDCl_3$) *S*-(4-acetylphenyl) thioacetate (2e)

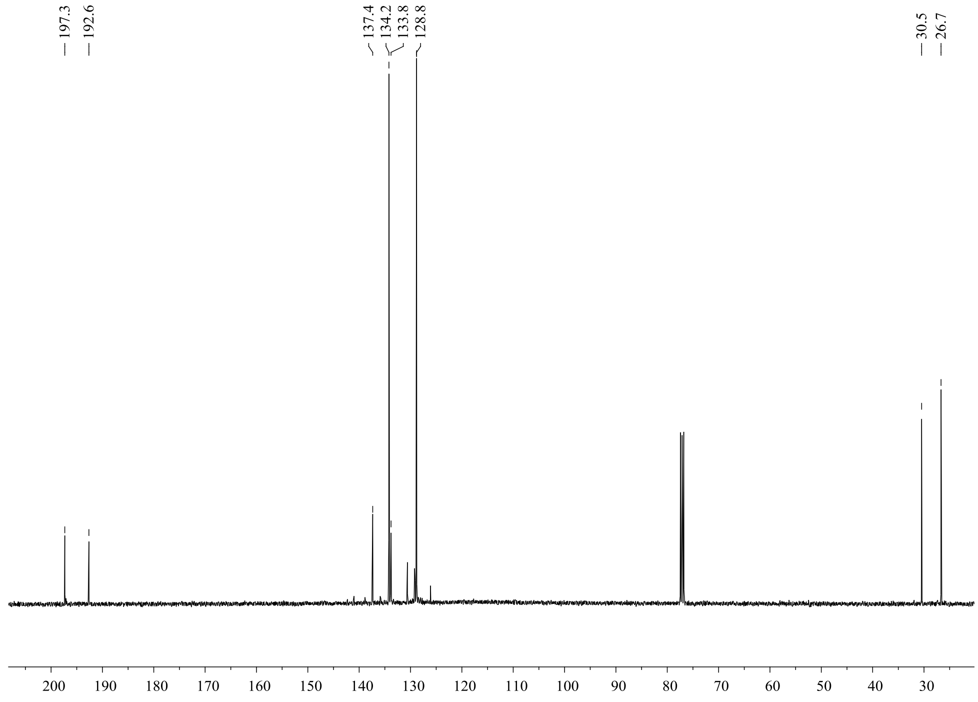

$^{1}H/^{13}C$ HSQC NMR ($CDCl_3$) *S*-(4-acetylphenyl) thioacetate (2e)

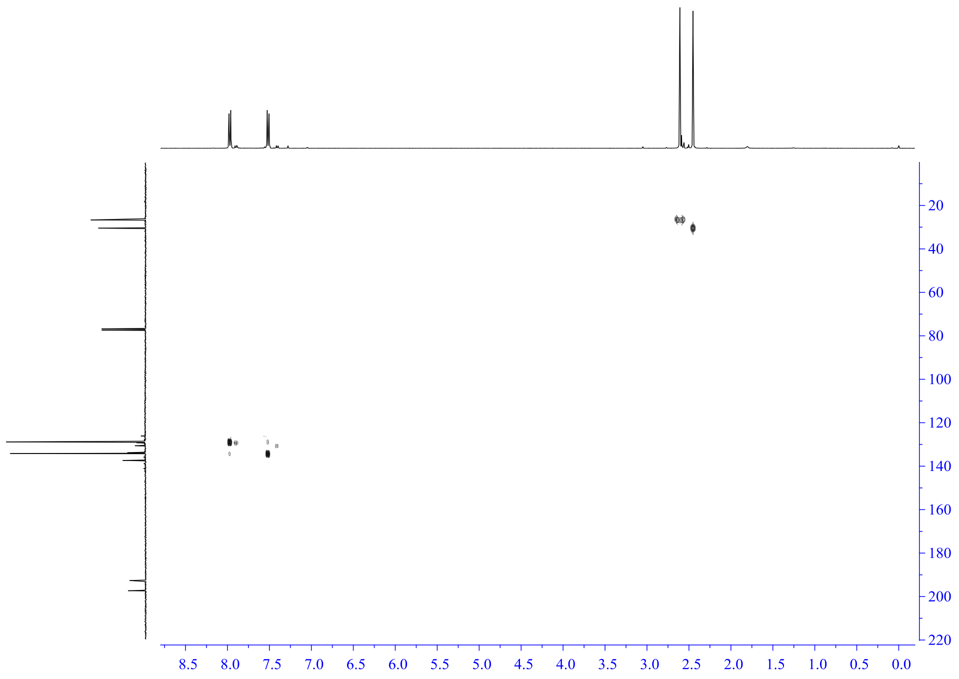

$^{1}H/^{13}C$ HMBC NMR ($CDCl_3$) *S*-(4-acetylphenyl) thioacetate (2e)

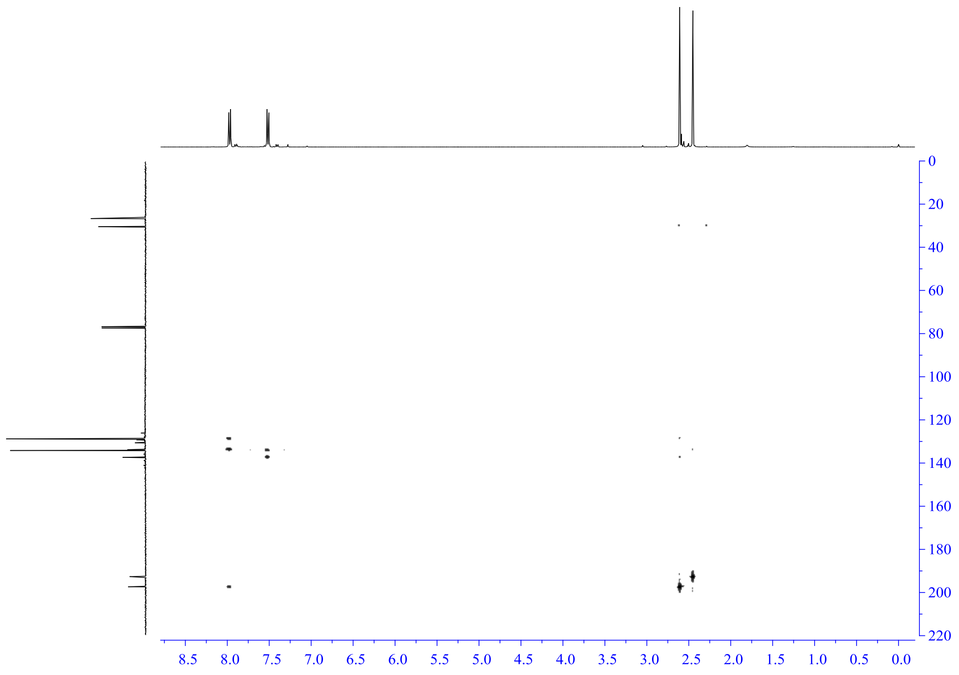

^{1}H NMR (400 MHz, $CDCl_3$) *S*-(4-nitrophenyl) thioacetate (2f)

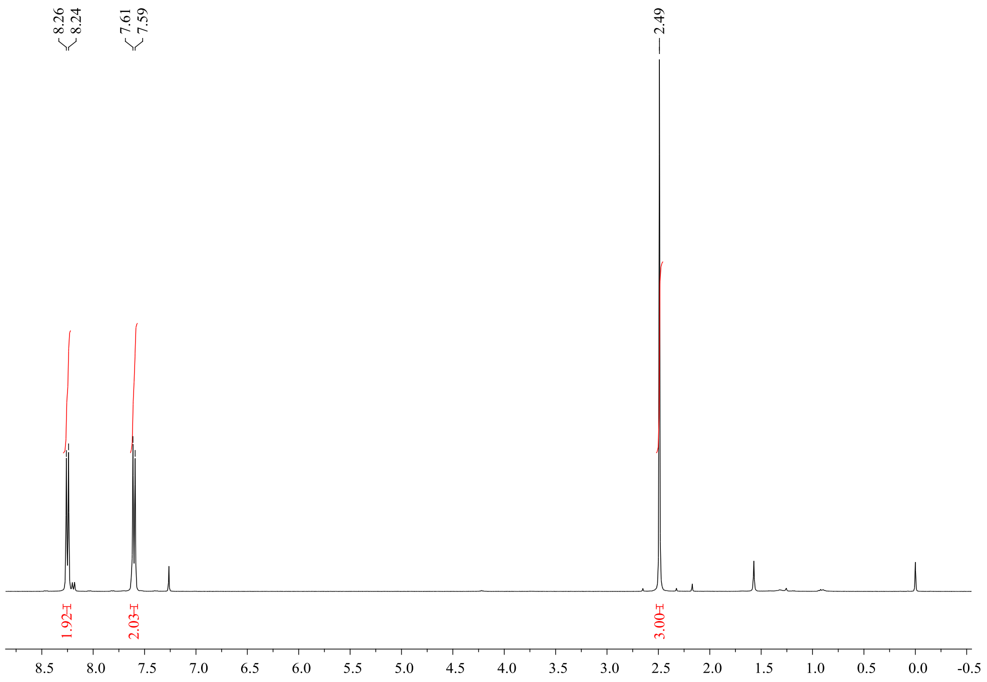

^{13}C NMR (101 MHz, $CDCl_3$) *S*-(4-nitrophenyl) thioacetate (2f)

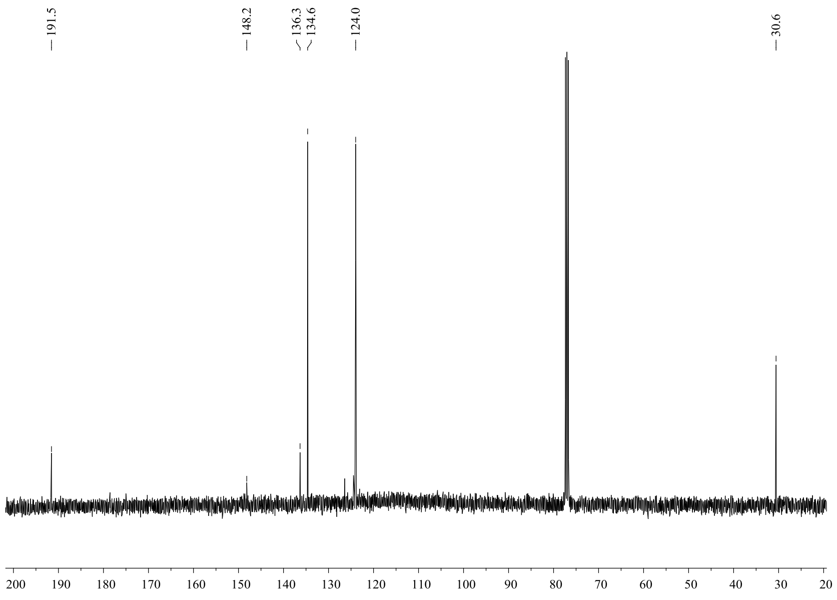

$^{1}H/^{13}C$ HSQC NMR ($CDCl_3$) *S*-(4-nitrophenyl) thioacetate (2f)

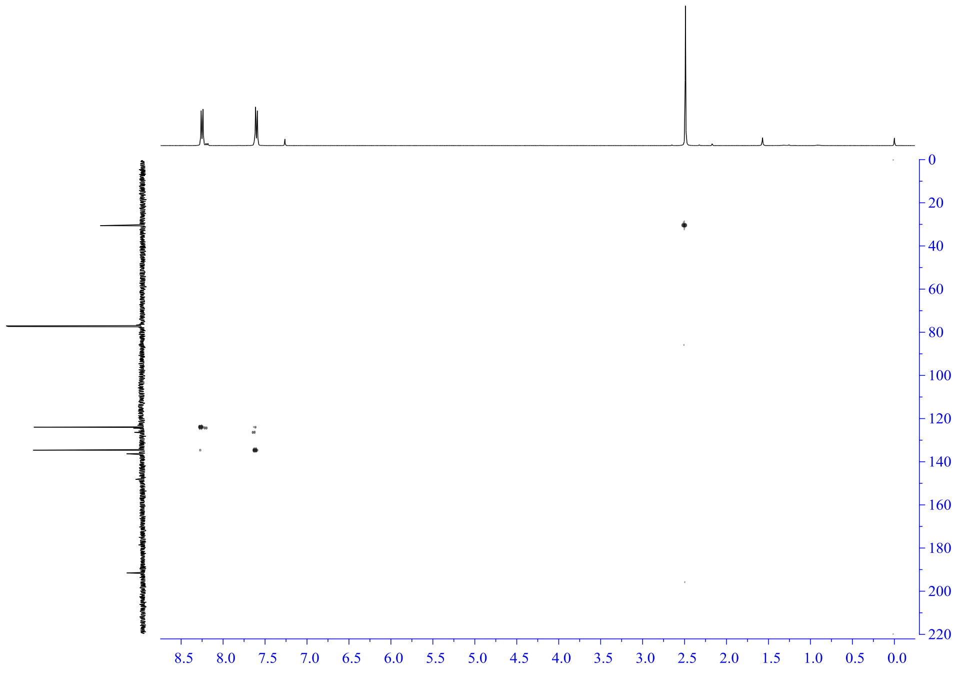

$^{1}H/^{13}C$ HMBC NMR ($CDCl_3$) *S*-(4-nitrophenyl) thioacetate (2f)

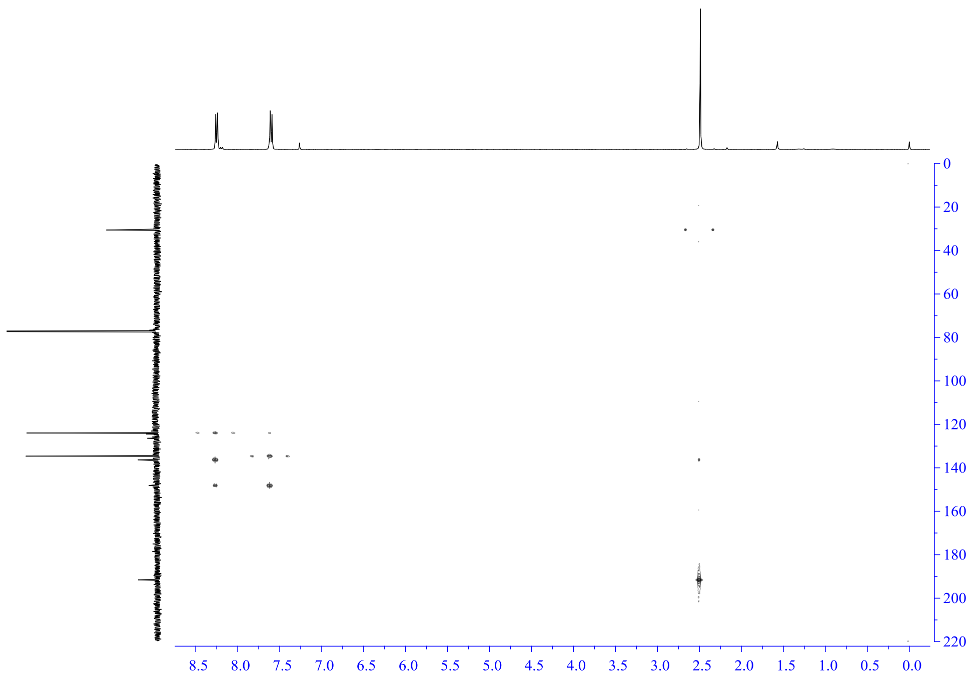

^{1}H NMR (400 MHz, $CDCl_3$) *S*-(4-cyanophenyl) thioacetate (2g)

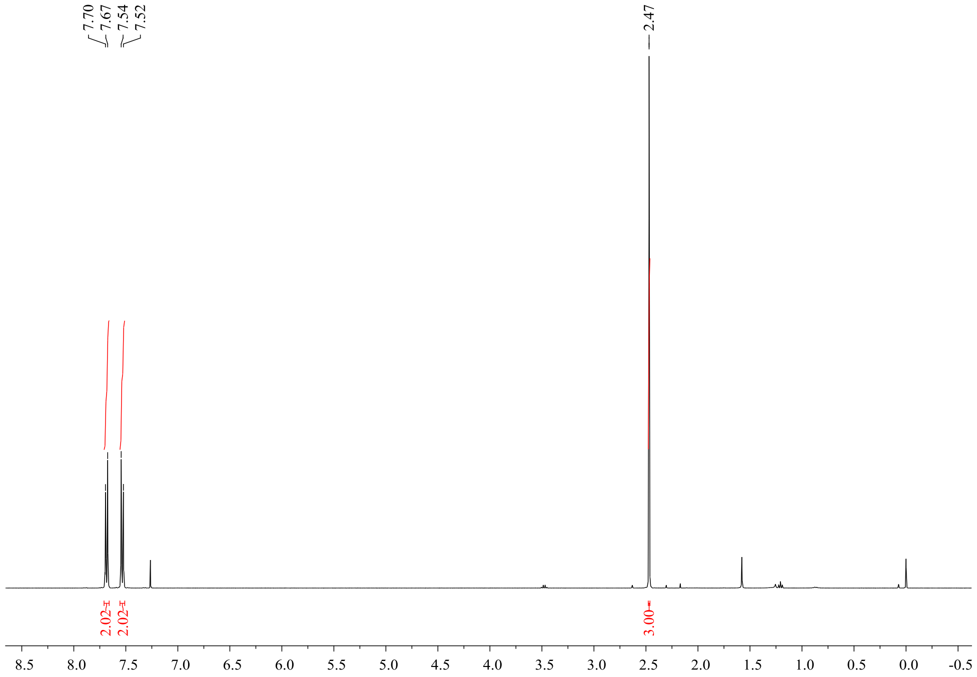

^{13}C NMR (101 MHz, $CDCl_3$) *S*-(4-cyanophenyl) thioacetate (2g)

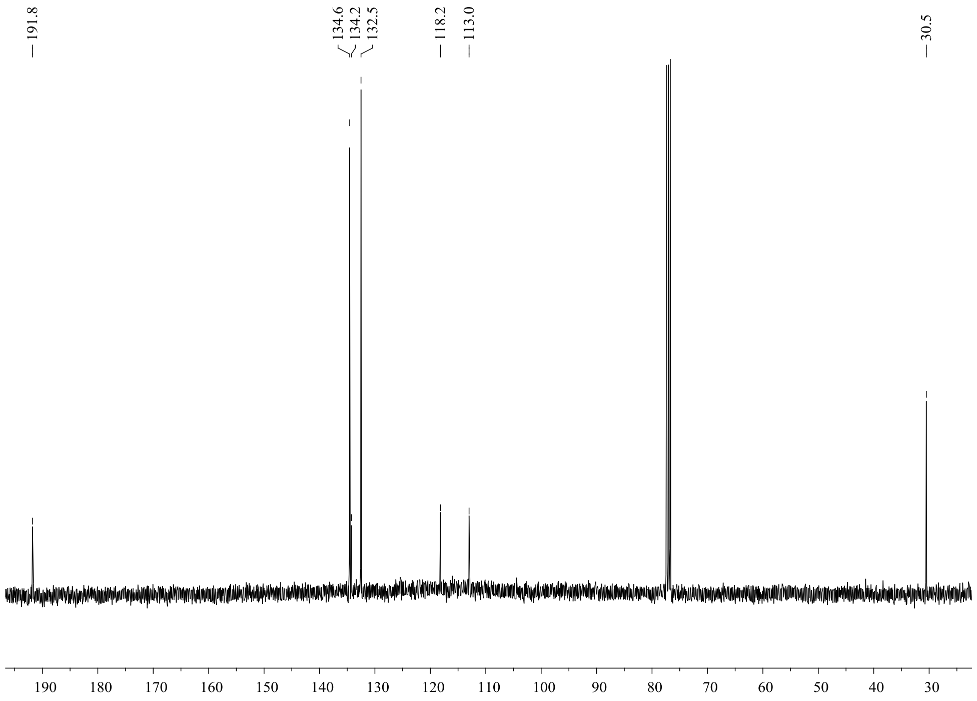

$^{1}H/^{13}C$ HSQC NMR ($CDCl_3$) *S*-(4-cyanophenyl) thioacetate (2g)

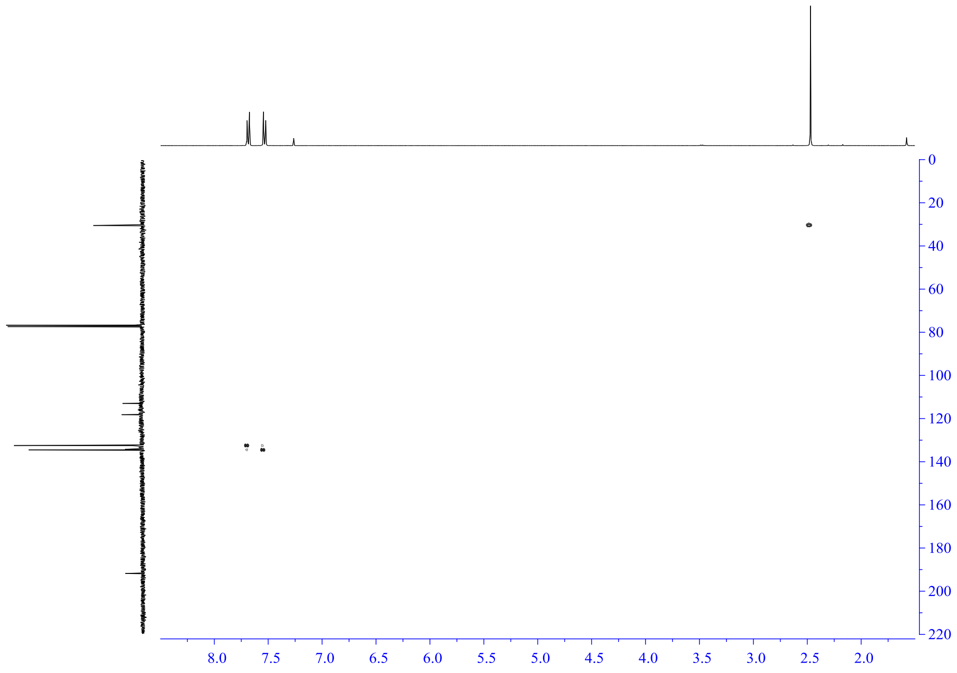

$^{1}H/^{13}C$ HMBC NMR ($CDCl_3$) *S*-(4-cyanophenyl) thioacetate (2g)

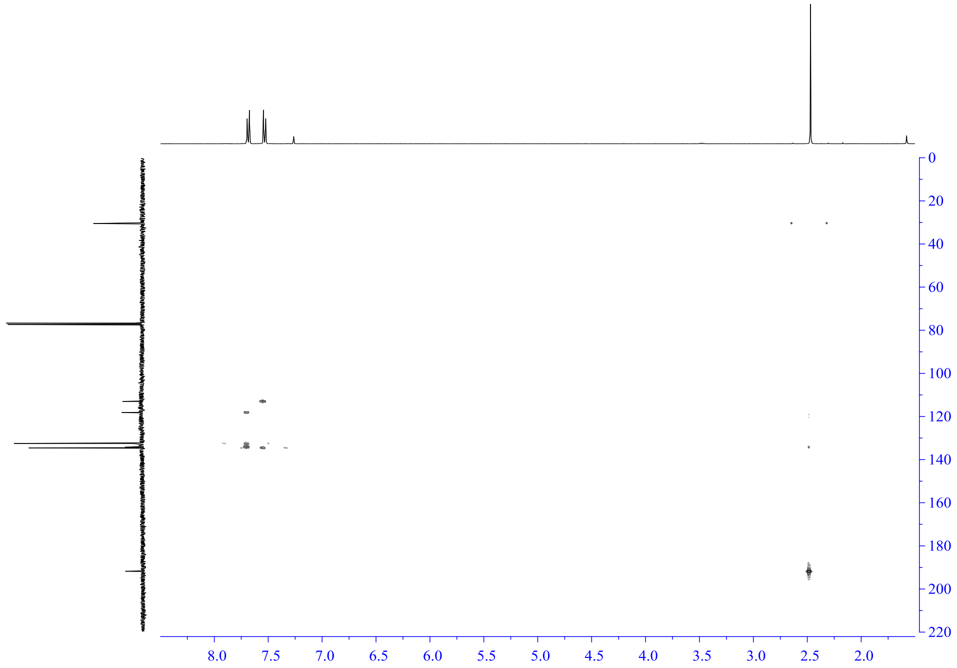

^1H NMR (400 MHz, $CDCl_3$) *S*-*o*-tolyl thioacetate (2h)

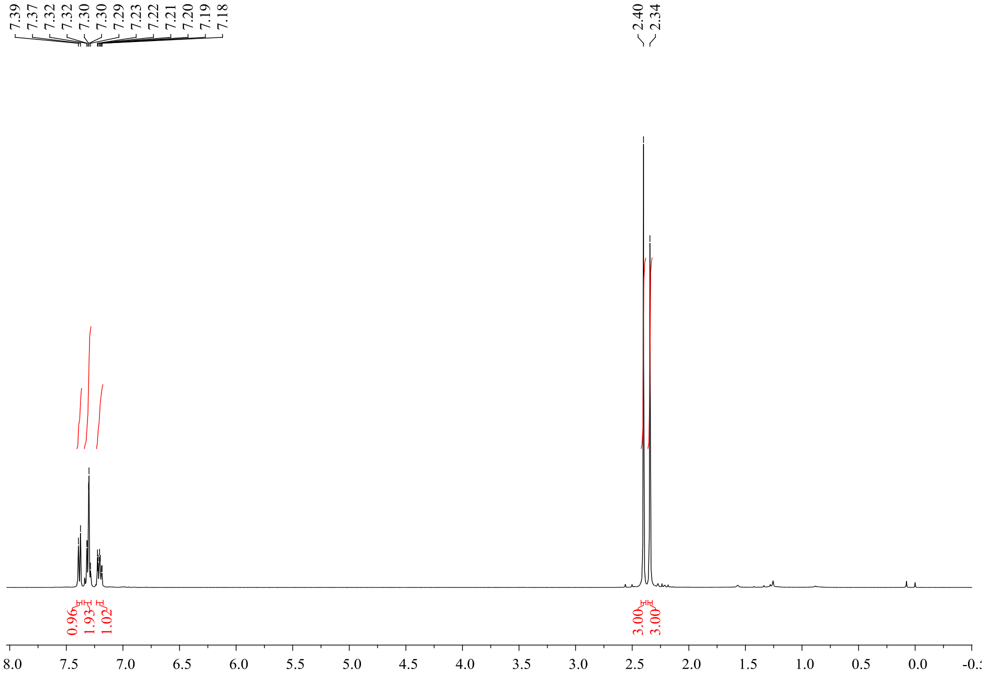

^{13}C NMR (101 MHz, $CDCl_3$) *S*-*o*-tolyl thioacetate (2h)

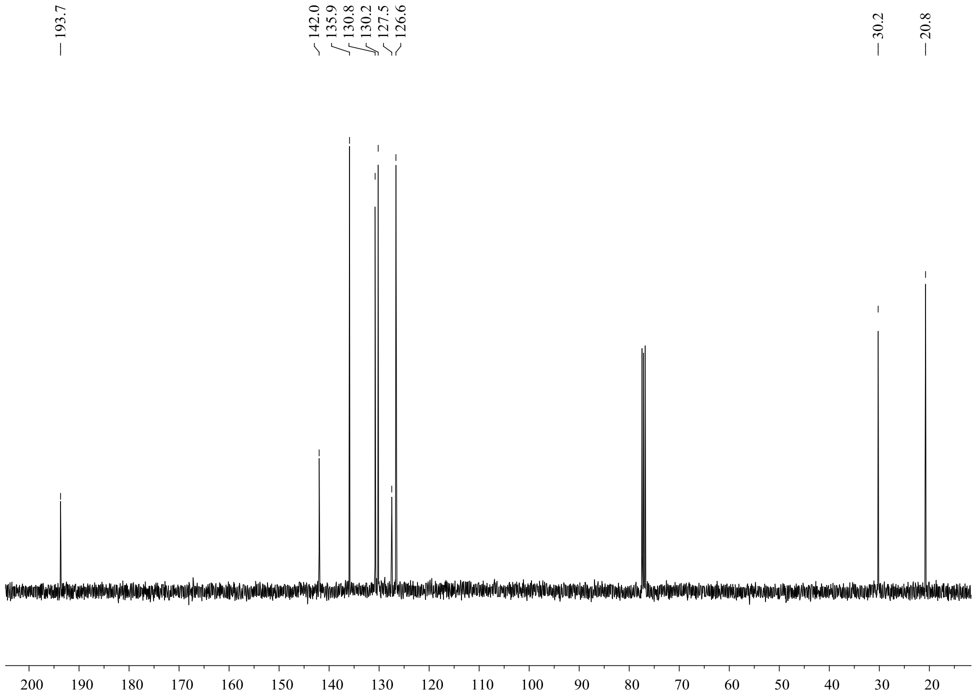

$^{1}H/^{13}C$ HSQC NMR ($CDCl_3$) *S*-*o*-tolyl thioacetate (2h)

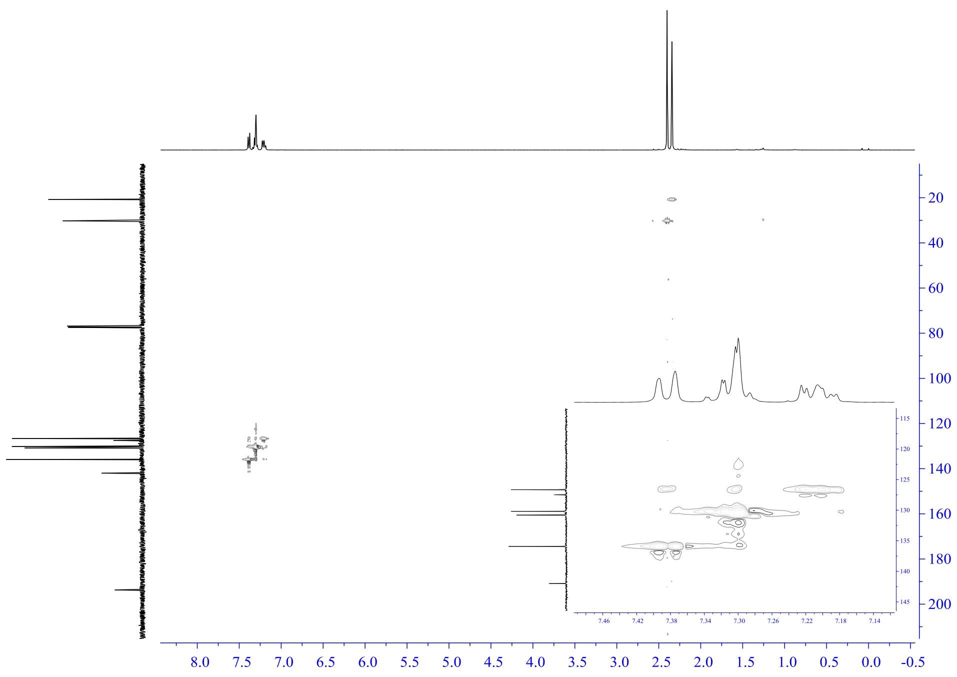

^{1}H NMR (400 MHz, $CDCl_3$) *S*-(2-methoxyphenyl) thioacetate (2i)

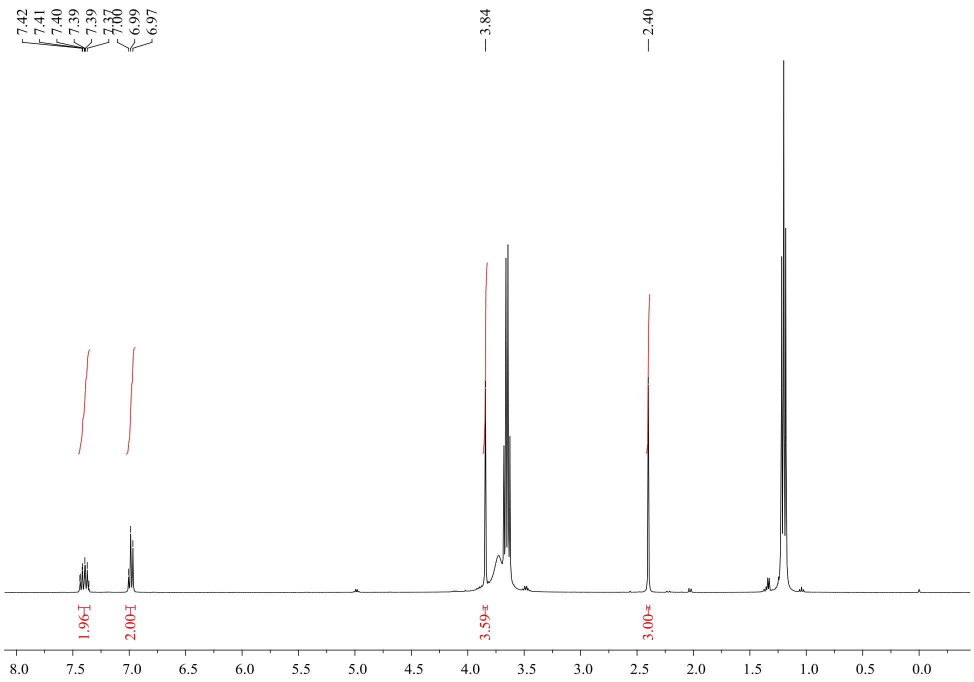

^{13}C NMR (101 MHz, $CDCl_3$) *S*-(2-methoxyphenyl) thioacetate (2i)

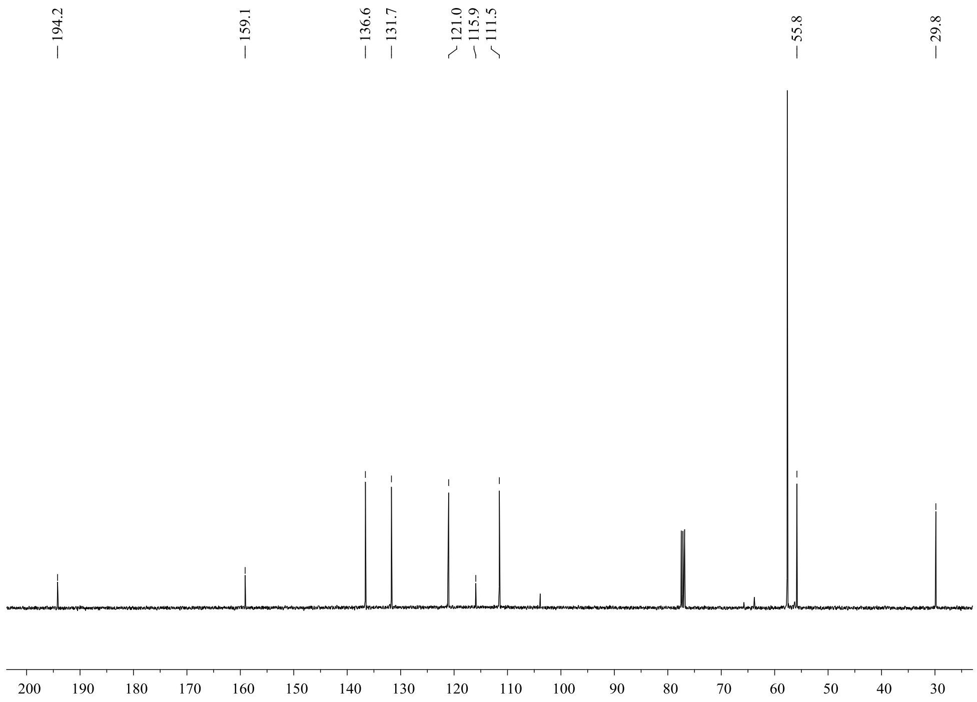

$^1H/^{13}C$ HSQC NMR ($CDCl_3$) *S*-(2-methoxyphenyl) thioacetate (2i)

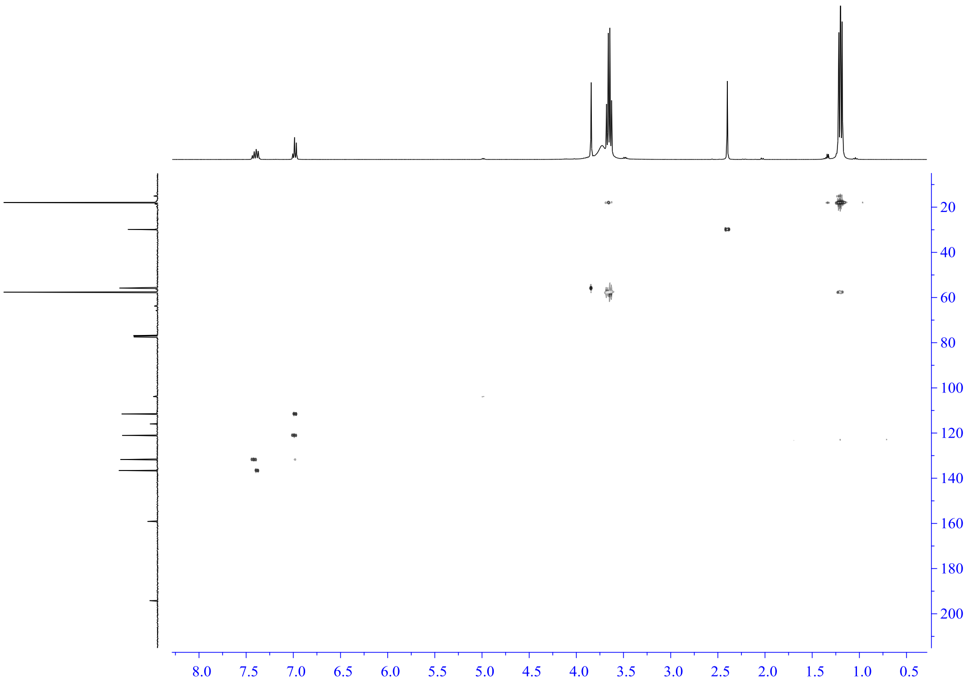

^{1}H NMR (400 MHz, $CDCl_3$) *S*-pyridin-3-yl thioacetate (3)

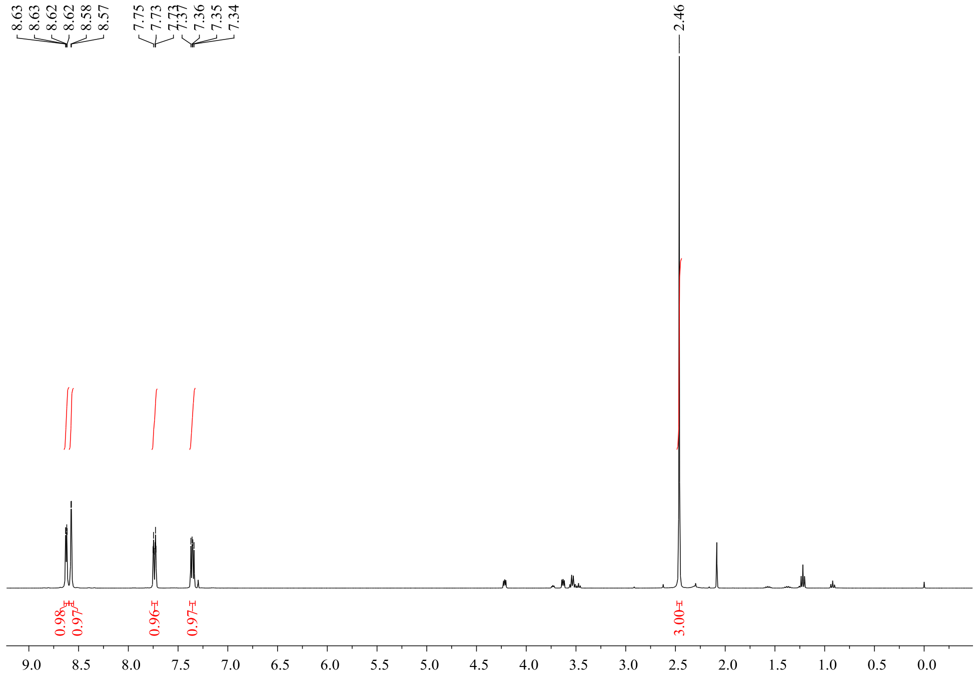

^{13}C NMR (101 MHz, $CDCl_3$) *S*-pyridin-3-yl thioacetate (3)

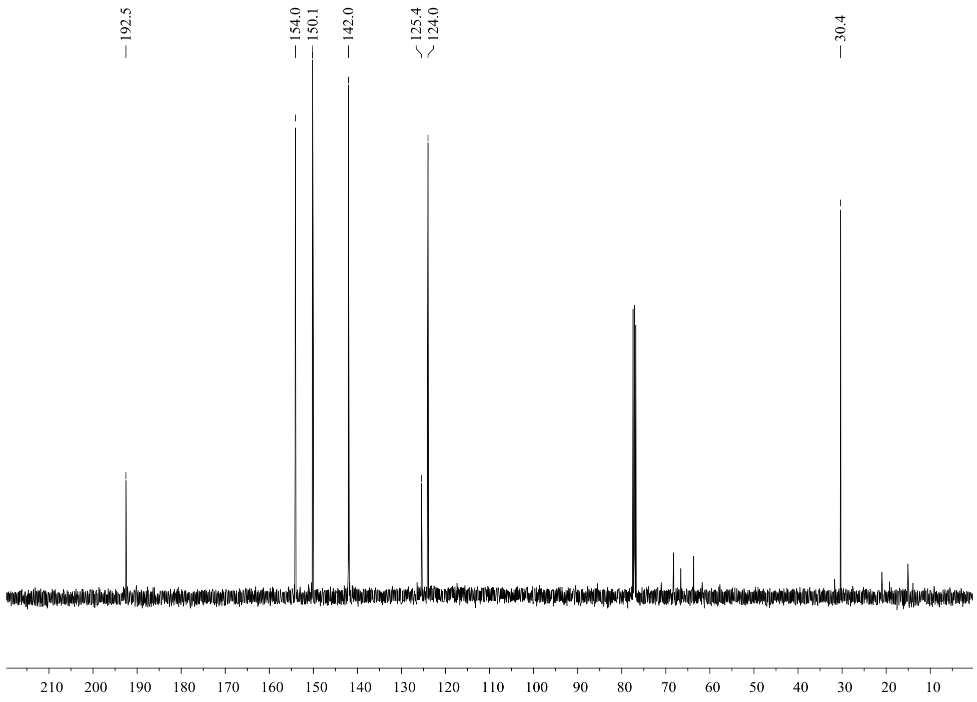

$^{1}H/^{13}C$ HSQC NMR ($CDCl_3$) *S*-pyridin-3-yl thioacetate (3)

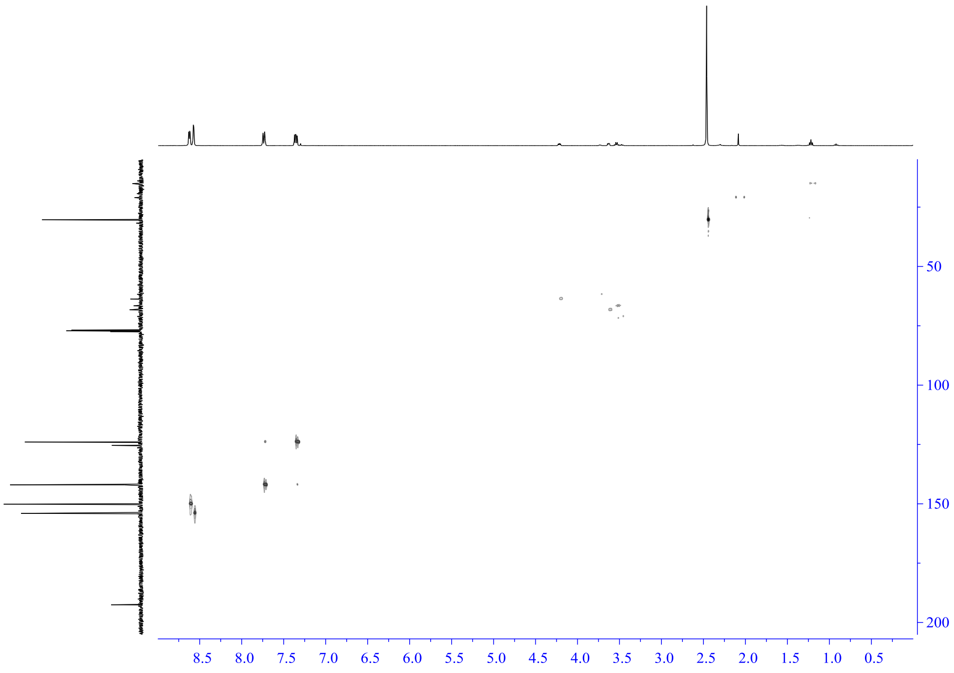

$^{1}H/^{13}C$ HMBC NMR ($CDCl_3$) *S*-pyridin-3-yl thioacetate (3)

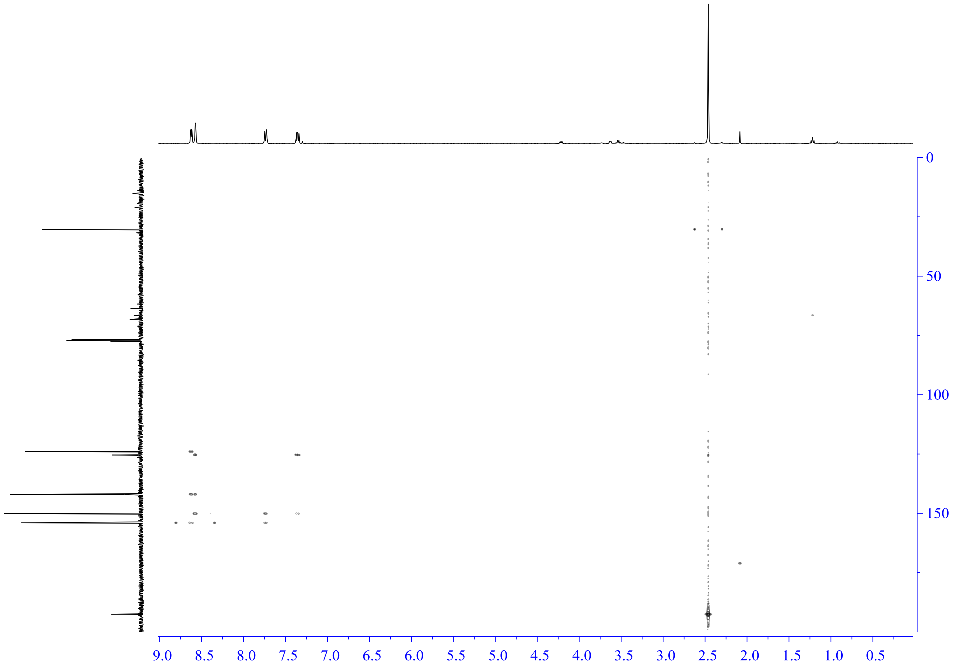

^{1}H NMR (400 MHz, $CDCl_3$) *S*-naphthalen-1-yl thioacetate (4)

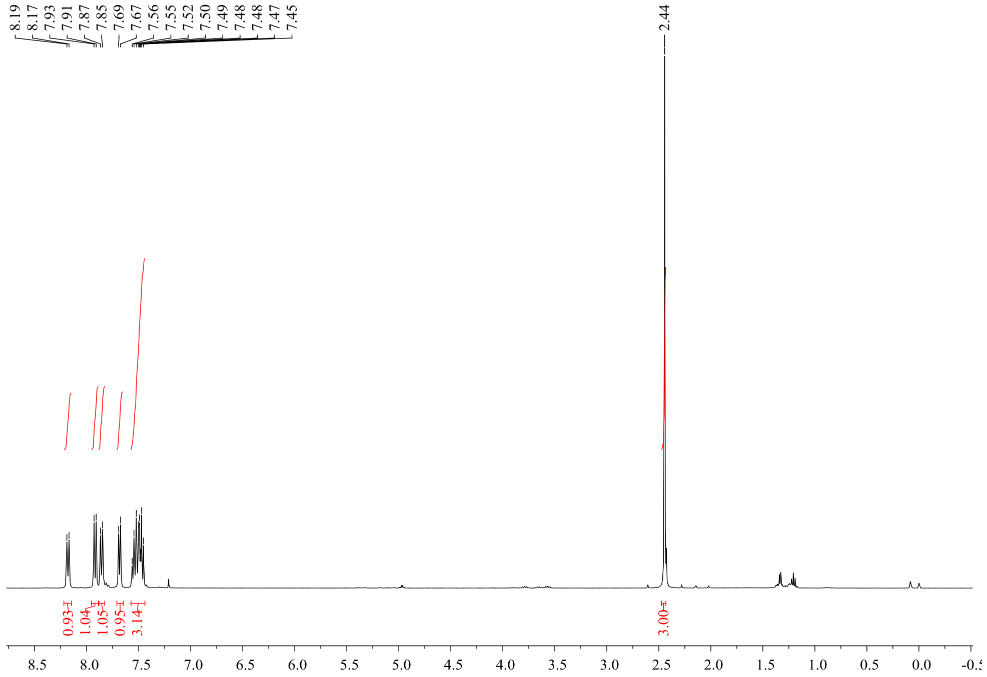

^{13}C NMR (101 MHz, $CDCl_3$) *S*-naphthalen-1-yl thioacetate (4)

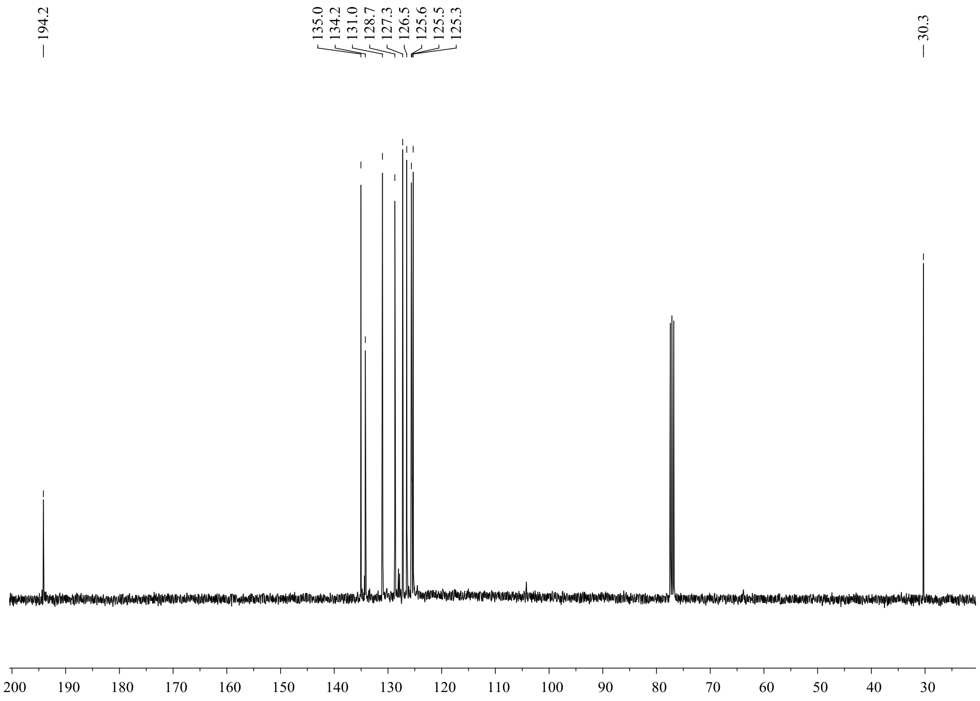

^{1}H/^{13}C HSQC NMR ($CDCl_3$) *S*-naphthalen-1-yl thioacetate (4)

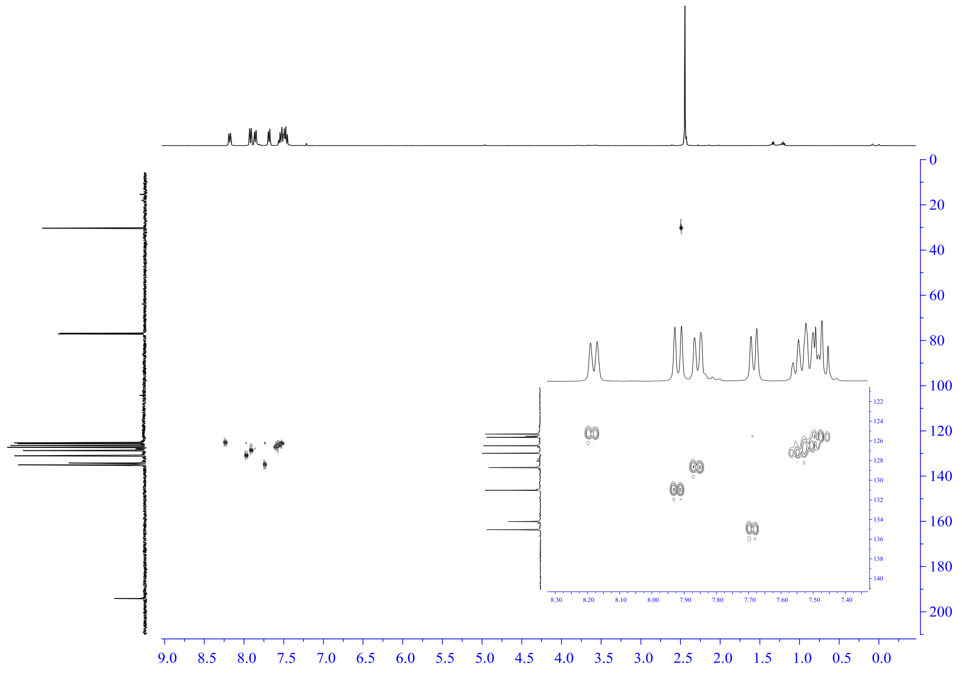

^{1}H NMR (400 MHz, $CDCl_3$) *S,S*'-1,4-phenylene dithioacetate (5)

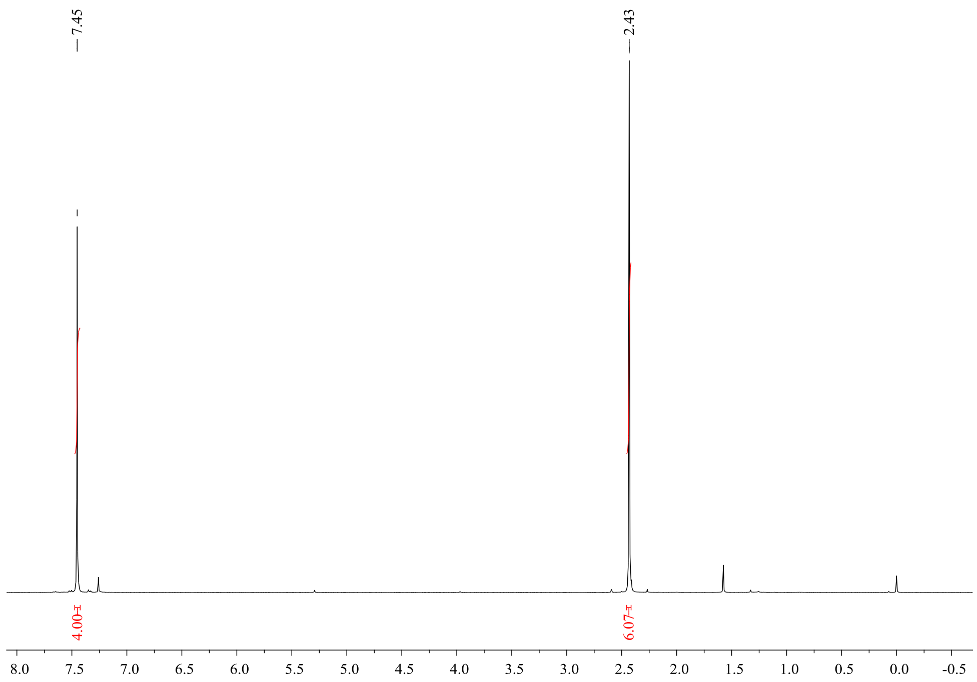

^{13}C NMR (101 MHz, CDCl$_3$) *S*,*S*'-1,4-phenylene dithioacetate (5)

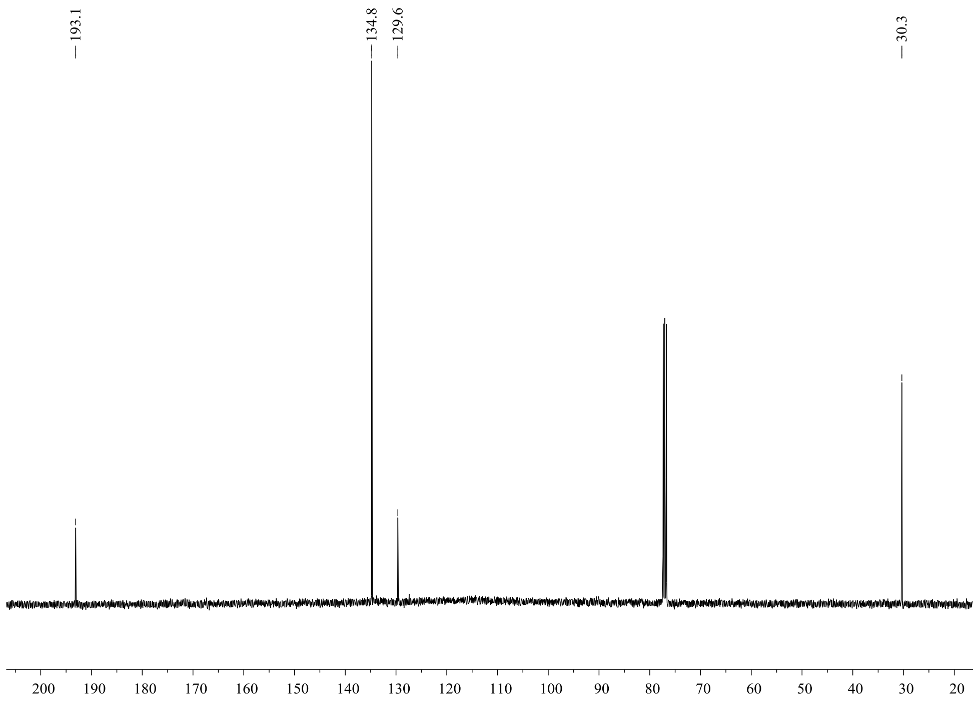

^{1}H NMR (400 MHz, CDCl$_3$) 3*H*-benzo[*c*][1,2]dithiol-3-one (6)

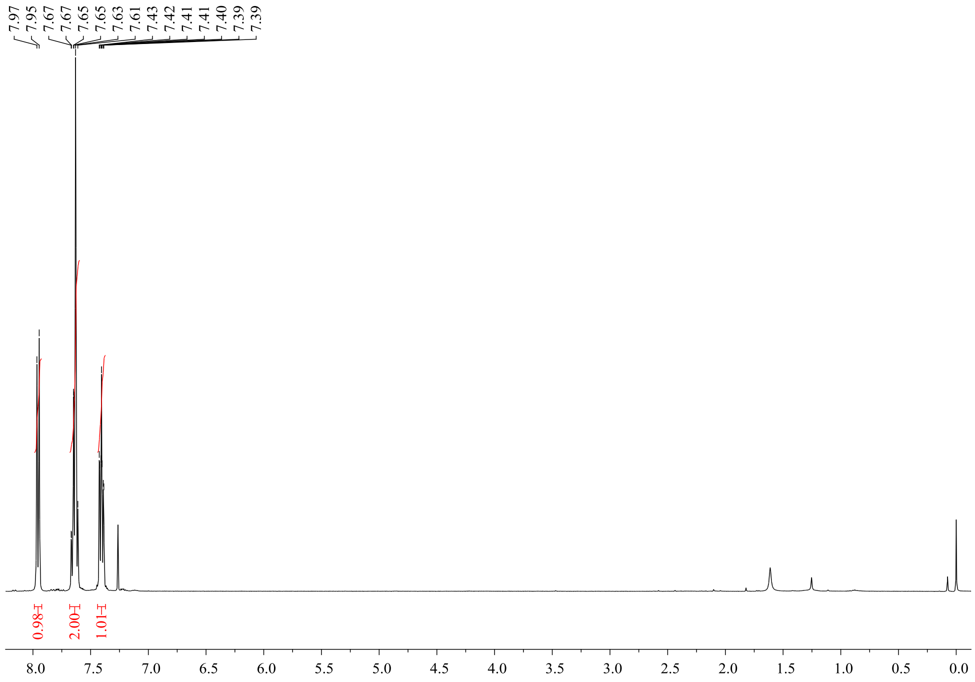

^{13}C NMR (101 MHz, $CDCl_3$) 3*H*-benzo[*c*][1,2]dithiol-3-one (6)

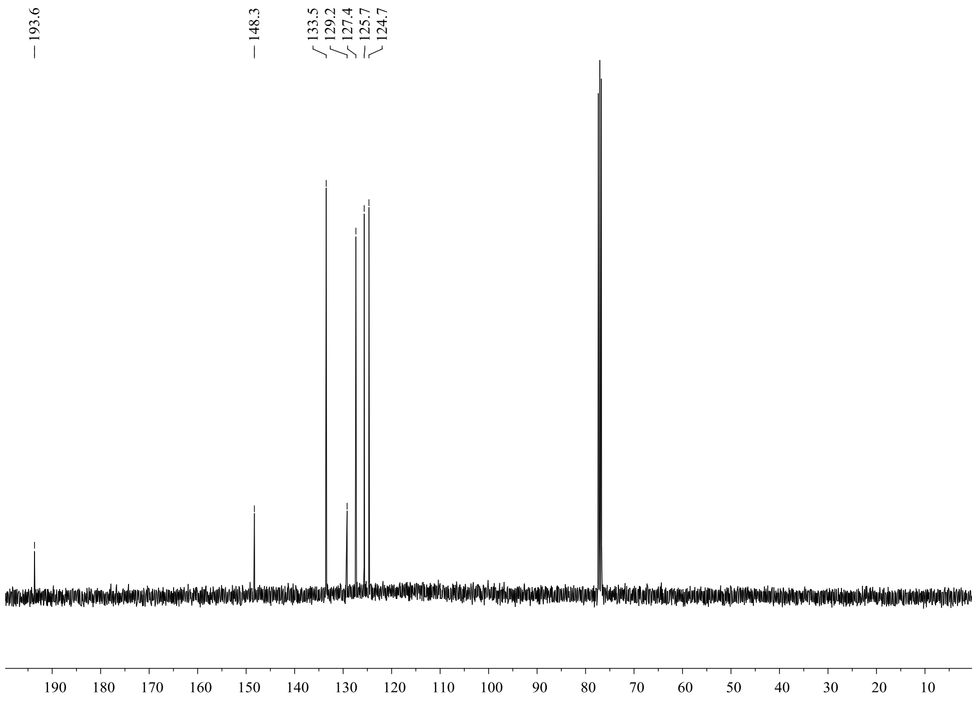

^{1}H/^{13}C HSQC NMR ($CDCl_3$) 3*H*-benzo[*c*][1,2]dithiol-3-one (6)

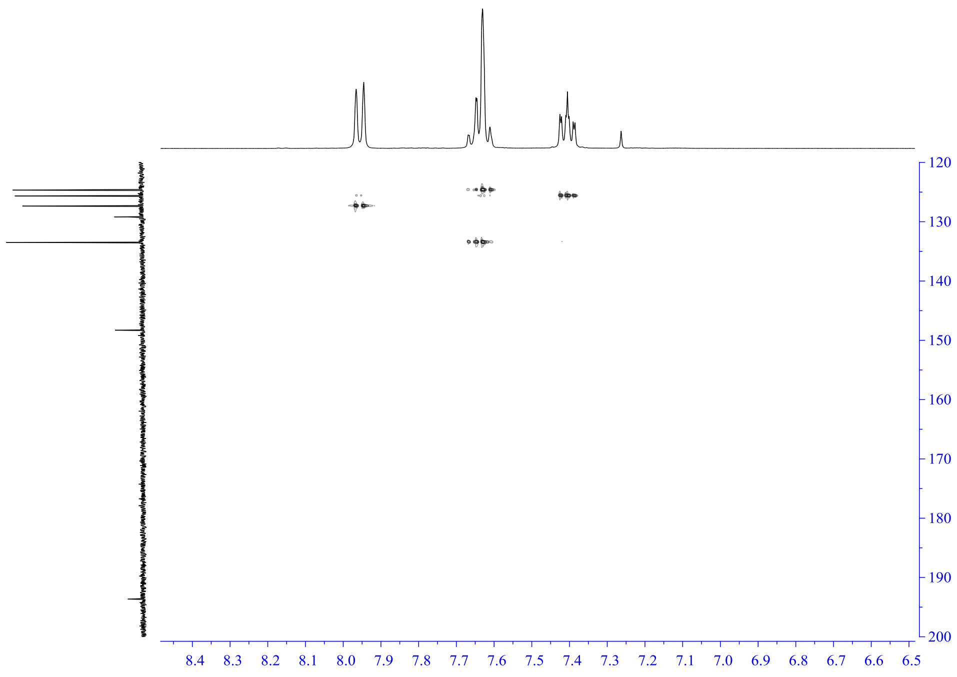

^{1}H NMR (400 MHz, $CDCl_3$) 2-mercaptobenzoic acid (7)

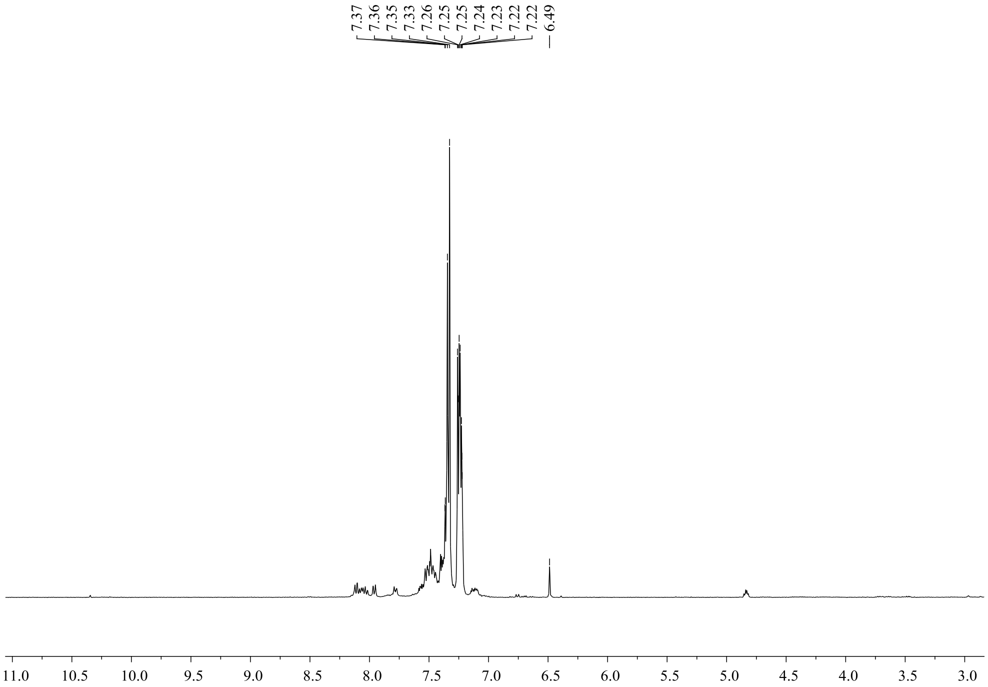

^{13}C NMR (101 MHz, $CDCl_3$) 2-mercaptobenzoic acid (7)

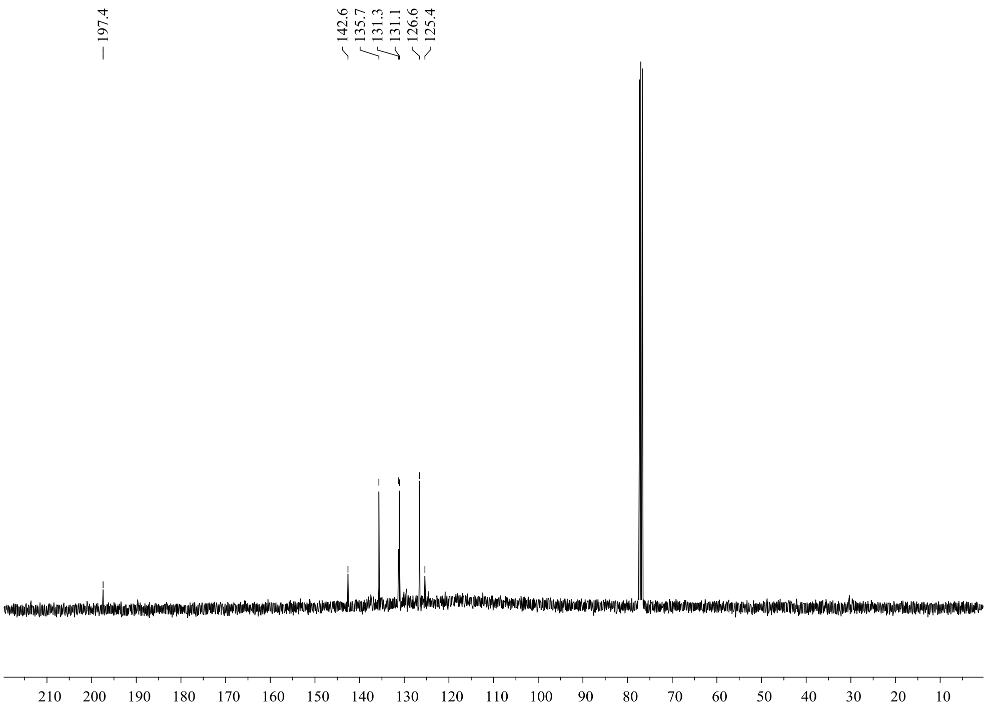

^{1}H NMR (400 MHz, $CDCl_3$) 2-methylbenzothiazole (14)

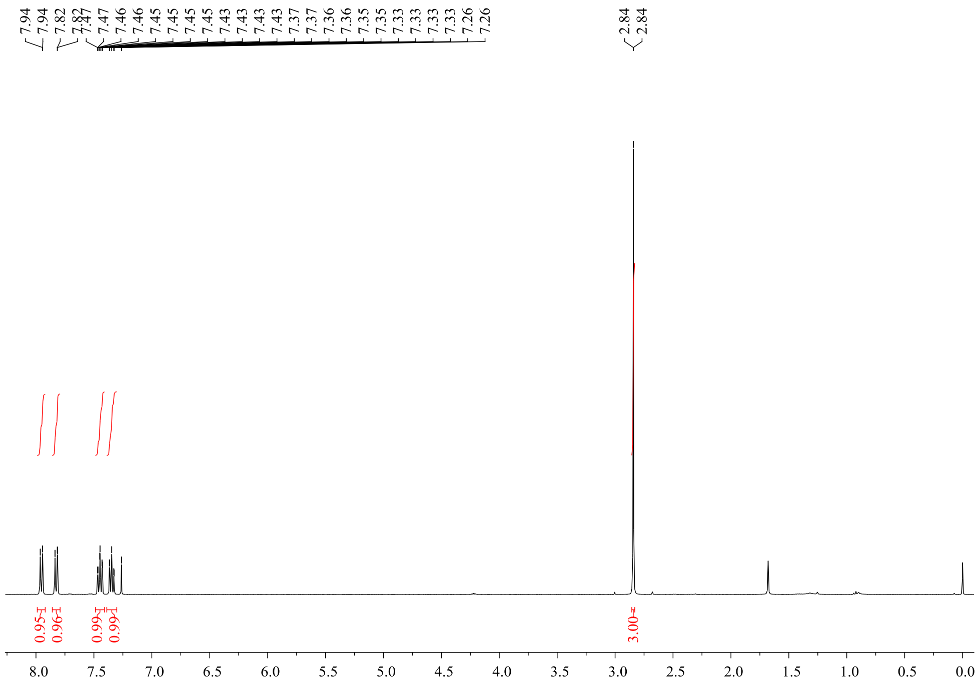

^{13}C NMR (101 MHz, $CDCl_3$) 2-methylbenzothiazole (14)

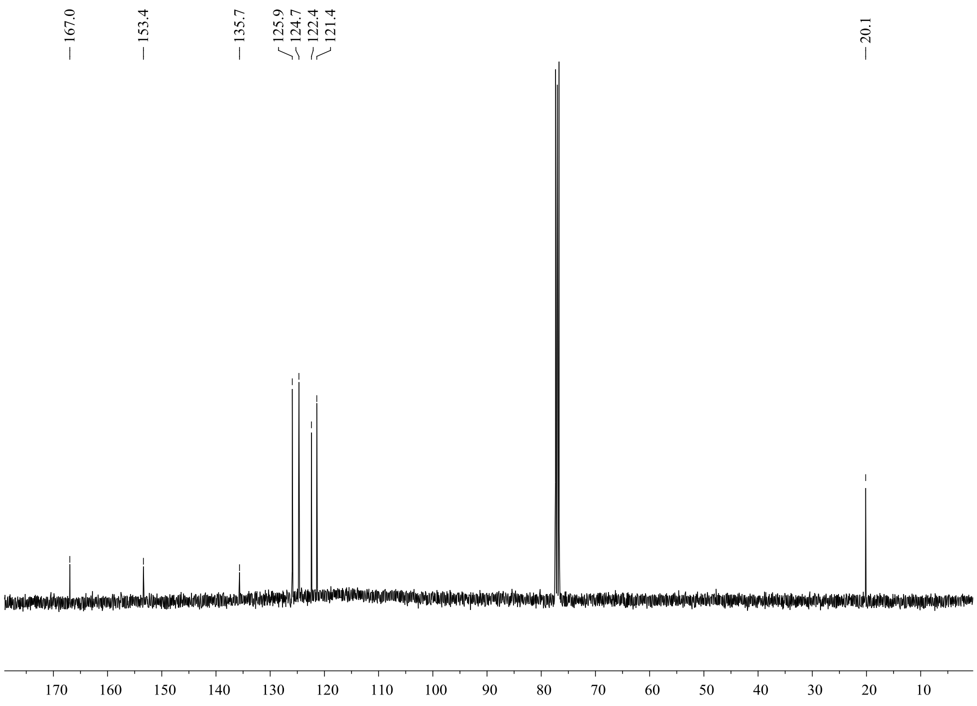

$^{1}H/^{1}H$ COSY NMR ($CDCl_3$) 2- methylbenzothiazole (14)

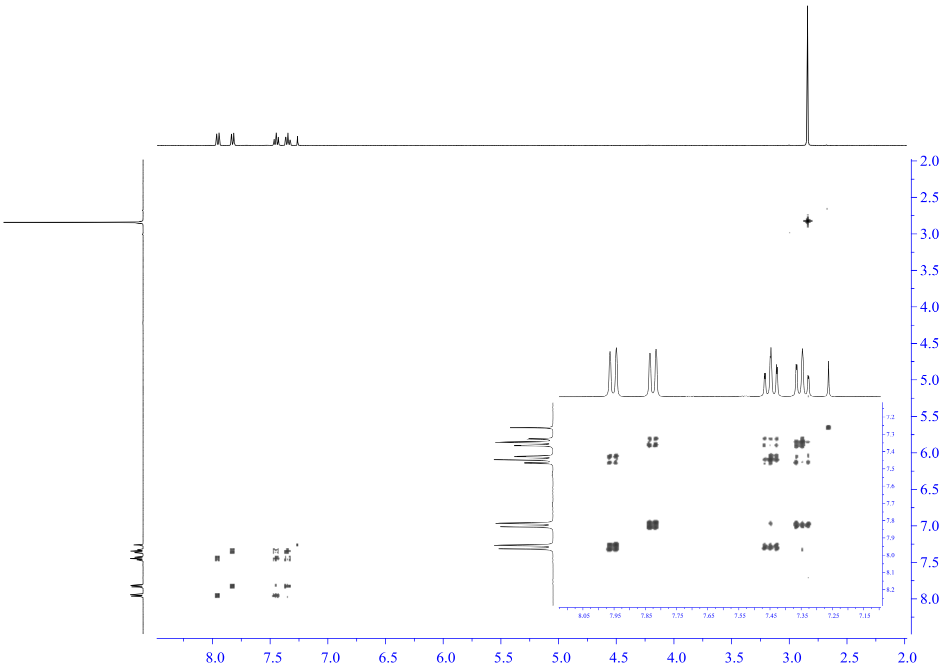

$^{1}H/^{1}H$ HSQC NMR ($CDCl_3$) 2- methylbenzothiazole (14)

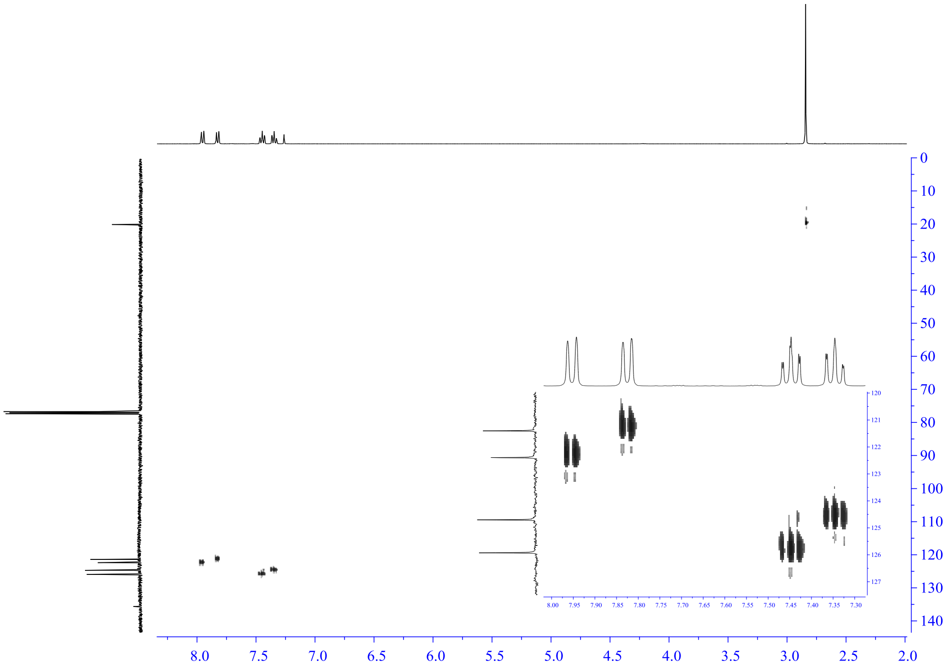

$^{1}H/^{13}C$ HMBC NMR ($CDCl_3$) 2- methylbenzothiazole (14)

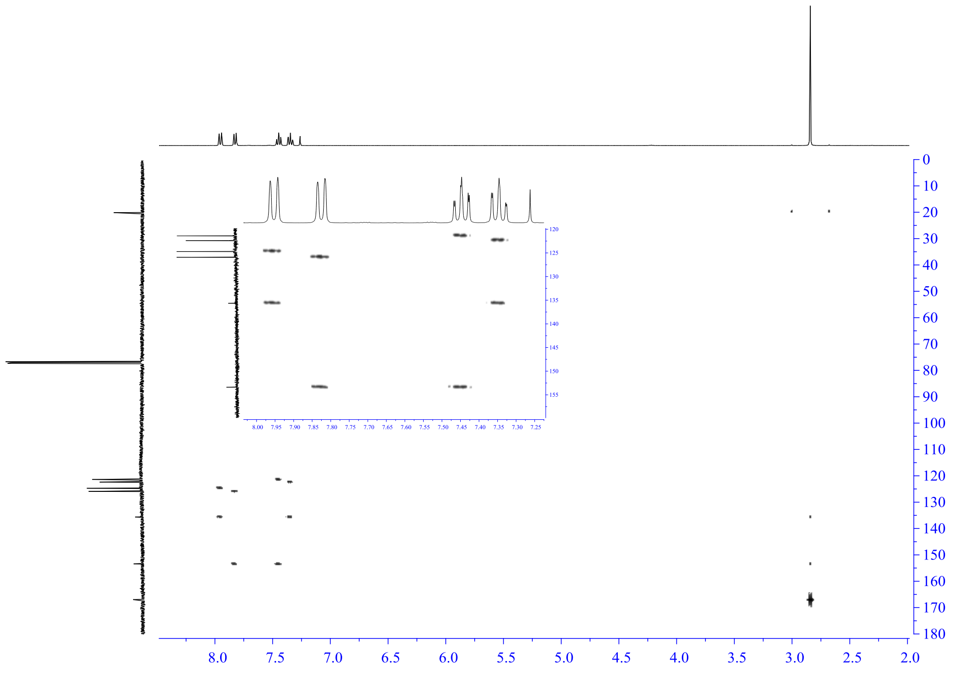

Supplement: File 1 — Experimental details, characterization data and spectra (1H, 13C NMR, and HSQC or HMBC as consigned) for all the products (2a–i, 3–7, and 14). [file Beilstein_J_Org_Chem-09-467-s001.pdf]
